# Expression Profiles of Long Noncoding RNAs in Intranasal LPS-Mediated Alzheimer's Disease Model in Mice

**DOI:** 10.1155/2019/9642589

**Published:** 2019-01-21

**Authors:** Liang Tang, Lan Liu, Guangyi Li, Pengcheng Jiang, Yan Wang, Jianming Li

**Affiliations:** ^1^Department of Human Anatomy, Histology and Embryology, Institute of Neuroscience, Changsha Medical University, Changsha, China; ^2^Department of Human Anatomy, School of Basic Medical Science, Changsha Medical University, Changsha, China; ^3^Medical College, Tibet University, Lhasa, China; ^4^Department of Neurology, Xiang-ya Hospital, Central South University, Changsha, China

## Abstract

Alzheimer's disease (AD), characterized by memory loss, cognitive decline, and dementia, is a progressive neurodegenerative disease. Although the long noncoding RNAs (lncRNAs) have recently been identified to play a role in the pathogenesis of AD, the specific effects of lncRNAs in AD remain unclear. In present study, we have investigated the expression profiles of lncRNAs in hippocampal of intranasal LPS-mediated Alzheimer's disease models in mice by microarray method. A total of 395 lncRNAs and 123 mRNAs was detected to express differently in AD models and controls (>2.0 folds,* p*<0.05). The microarray expression was validated by Quantitative Real-Time-PCR (qRT-PCR). The pathway analysis showed the mRNAs that correlated with lncRNAs were involved in inflammation, apoptosis, and nervous system related pathways. The lncRNA-TFs network analysis suggested the lncRNAs were mostly regulated by HMGA2, ONECUT2, FOXO1, and CDC5L. Additionally, lncRNA-target-TFs network analysis indicated the FOXL1, CDC5L, ONECUT2, and CDX1 to be the TFs most likely to regulate the production of these lncRNAs. This is the first study to investigate lncRNAs expression pattern in intranasal LPS-mediated Alzheimer's disease model in mice. And these results may facilitate the understanding of the pathogenesis of AD targeting lncRNAs.

## 1. Introduction

Alzheimer's disease (AD), with principal clinical manifestations of memory loss, cognitive decline, and dementia, is a common progressive neurodegenerative disease in aging people worldwide [1, 2]. The main neuropathic characteristics of AD are marked by extracellular amyloid-*β* (A*β*) deposition, neurofibrillary tangles (NFTs), loss of neuron, and synaptic and dystrophic neuritis [3–5]. The pathogenesis of AD is largely unknown. Multiple factors such as genetics, free radical injury, apoptosis, and inflammation were considered to be involved in the development of AD [6–8]. Recently, many susceptible genes including BACE1 [9], PS1/2 [10], APP [11], APOE [12], and SORL1 [13] have been found to be associated with the AD risk. However, the recent associated-genes could not explain the whole pathogenesis of AD.

Recent genomic studies have investigated thousands of noncoding RNAs (ncRNA) in both animal models and human beings. The long noncoding RNAs (lncRNAs), defined as the transcripts of >200bp, was considered to be one of the most important ncRNAs. Increasing evidence has indicated that lncRNAs may participate in multiple essential biological processes such as genomic imprinting, immune response, and disease development [14–16]. In the last decade, the role of lncRNAs in AD has gained considerable attention and has been investigated by a multitude of studies. Certain lncRNAs such as BACE1-AS [17], 51A [18], 17A [19], NDM29 [20], BC200 [21], and NAT-Rad18 [22] have been identified in human brain tissues with AD. Moreover, the expression profiles of lncRNAs in AD patients [23, 24], transgenic AD mice model [25], and AD rat model [26] have been investigated. Although many studies on the expression profiles of lncRNAs in AD models and human beings have been performed, the knowledge of the expression patterns and potential biological functions of lncRNAs in AD remains to be far from clear.

In the present study, we have investigated the different expression profiles of AD-related lncRNAs and mRNAs by using microarray analysis in the hippocampus of intranasal LPS-mediated mice model, as well as matched controls. The results were identified by qRT-PCR. And the gene ontology (GO), Kyoto Encyclopedia of Genes and Genomes (KEGG), coexpression of lncRNAs-mRNAs network, potential lncRNA-TF (transcription factors) network, and lncRNA-target-TFs network were analyzed.

## 2. Materials and Methods

### 2.1. Animals and Study Design

The homozygous male C57BL/6J mice, 20±5g, were purchased from Hunan slack scene of laboratory animal Co., Ltd. The protocols of these animals followed the National Institutes of Health Guide for the Care and Use of Laboratory Animals. And the research procedures were approved by the Ethics Committee of the Changsha Medical University, China. The mice were housed in a room with the temperature of 22±0.8°C, 50±10% relative humidity, 12h light/dark cycle, and free to water and food. A total of 20 mice were randomly divided into two groups (AD group: n=10, control group: n=10). The AD group was treated with intranasal LPS 10ul (right side, 1mg/ml), while the control group was treated with intranasal saline 10ul (right side, 0.9%) with the treatment duration of 5 months.

### 2.2. Morris Water Maze Test

The Morris water maze test was conducted to evaluate the change of spatial learning and memory deficits at the last six day in each month (five consecutive days of escape training and one day of probe trail). The test protocol followed a previously published study by Vorhees et al [27]. The trials and movements tracking of the animals were recorded by the ANY-maze video tracking system (Stoelting Co., USA). The swim paths, escape latency, and the frequency of crossing the target platform were recorded and analyzed.

### 2.3. Sample Collection

The mice were anesthetized with pentobarbital sodium (0.2%, 0.1ml/10g) by intraperitoneal injection. The cerebrospinal fluid (CSF) was collected by puncturing the cerebellomedullary cistern. And the peripheral blood (PB) was obtained by removing the eyeball. All the samples were stored at 4°C. In addition, the hippocampal tissues and whole brains were stored at -80°C until further analysis.

### 2.4. ELISA

The level of proinflammatory cytokines including interleukin-6 (IL6), tumor necrosis factor- (TNF-) *α*, IL1-*β*, and IL10 in both CSF and PB were measured by enzyme-linked immunosorbent assay (ELISA). The ELISA kits were purchased from Shanghai Beinuo Bio Co., Ltd. The microplate spectrophotometer (Multiskan MK3, Finland) was applied to detect the proinflammatory cytokines. The data is represented by mean ± standard deviation (mean ± SD). The* t*-test was applied in the intergroup comparisons (AD: CSF and PB; AD and controls). Linear correlation analysis was used.* P* < 0.05 was considered statistically significant. SPSS 15.0 were applied to carry out statistical analysis.

### 2.5. Immunohistochemistry

Three hippocampal tissues of AD models and controls were disposed in parallel. Tissues were first treated free-floating with 5% H_2_O_2_ in PBS for 30 min and 5% normal bovine serum in PBS with 0.3% Triton X-100 for 1h to lower nonspecific reactivity. The tissues were first incubated overnight with mouse anti-GFAP (1:300; Chemicon, USA) at 4°C, and then reacted with bovine anti-mouse immunoglobulin G (IgG) (1:400; Chemicon, USA) for 1h, incubated with avidin-biotin complex reagents (1:400; Burlingame, CA, USA) at room temperature for 1h. The immunoreactive product was visualized in 0.003% H_2_O_2_ and 0.05% 3, 3'-diaminobenzidine. The results were detected by light microscope.

### 2.6. Western Blot Analysis

The frozen hippocampal tissues of AD models and controls were homogenized by sonication and centrifuged at 15,000 × g. Protein was collected from the supernatants and measured by BCA protein assay kit (Thermo Fisher Scientific). 50 *μ*g protein was separated with 15% sodium dodecyl sulfate-PAGE gels and then electrotransferred onto polyvinylidene fluoride membranes (Millipore, Shanghai Trading Company Ltd., Shanghai, China). Separated protein was then immunoblotted with mouse anti-GFAP (1:400; Wuhan Servicebio Technology Co., Ltd., Wuhan, China) and mouse anti-GAPDH (1:5000; Wuhan Servicebio Technology Co., Ltd., Wuhan, China). The membranes further reacted with anti-mouse IgG (1:20,000; Thermo Fisher Scientific). Immunoblot signaling was visualized with the Pierce ECL-Plus Western Blotting Substrate detection kit (Thermo Fisher Scientific), followed by X-ray film exposure and image capture in a laser scanner. Quantitative analysis of GFAP-positive dots was performed with Image-Pro Plus software.

### 2.7. RNA Extraction

The total RNA was isolated from each hippocampal tissue sample by using AMBION TRIZOL reagent kit (Invitrogen, Grand Island, NY, USA). The RNA quantity and integrity were measured by NanoDrop ND-1000 (Thermo Scientific) and Agilent 2100 bioanalyzer (Agilent Technologies).

### 2.8. Microarray Analysis

The total RNA was purified by RNasey Mini Kit (Qiagen p/n 74104). Then, fluorescent cRNA with Cyanine-3-CTP was prepared by Quick Amp Labeling Kit, One-Color (Agilent p/n 5190-2305). The labelled cRNAs were purified using an RNeasy Mini Kit (Qiagen p/n 74104). The labeled cRNA was hybridized onto the microarray using an Agilent Gene Expression Hybridization Kit (Agilent p/n 5188-5242). And then the hybridized microarrays were washed, fixed, and scanned using an Agilent Microarray Scanner (Agilent p/n G2505C). Data were extracted using Feature Extraction Software (version 10.7.1.1, Agilent Technologies). All the experiments were carried out according to the manufacturer's standard protocols. The experiments were performed by OEBiotech Corporation (Shanghai, China). Agilent mouse lncRNA Microarray V3 (4*∗*180K, Design ID: 084388) was used in the present experiment.

### 2.9. Differential Expression Analysis

The data analysis was performed by Genespring (version 13.1, Agilent, Santa Clara, USA). All the raw data including original signal values, normalized signal values, and detailed annotation information were normalized by quantile method.* t*-test was used to screen differentially expressed lncRNAs and mRNAs. And a fold change>= 2.0 and a* P* value <= 0.05 between the compared two groups for lncRNAs or mRNAs was considered as differentially expressed. The hierarchical clustering of differentially expressed lncRNAs and mRNAs between AD and control hippocampal samples was also performed to display the distinguishable genes' expression pattern among samples by using the Genespring (version 13.1, Agilent, Santa Clara, USA). Afterwards, Gene Ontology enrichment analysis (GO) (http://www.geneontology.org) and Kyoto Encyclopedia of Genes and Genomes (KEGG) analysis (http://www.genome.jp/kegg/) were applied to determine the roles of these differentially expressed mRNAs.

### 2.10. Quantitative Real-Time-PCR (qRT-PCR)

The total RNA samples were purified with DNase. And the cDNA were synthesized by using a TIANscript RT Kit (Invitrogen, Grand Island, NY, USA). The qRT-PCR was performed using the SYBR Green Premix DimerEraser kit (TaKaRa Bio Inc., Dalian, China) on the Roche LightCycler 480 Instrument II. The relative expression levels of lncRNAs and mRNAs were analyzed using the 2^−ΔΔCt^ method and were normalized to GAPDH. Student's* t*-test was used to access the significance of differences. ANOVA was performed for repeated measures. A value of* p* < 0.05 was considered to be statistically significant. The statistical tests were performed using the SPSS (version 19.0, SPSS, Inc., Chicago, IL, USA). The primers of randomly selected lncRNA and mRNAs were listed in Supplementary File Table 1 (Table s1).

### 2.11. Coexpression Network Analysis

The normalized signal intensities of specific mRNA and lncRNA expression levels were used to construct coexpression network. Pearson's correlation coefficients (PCC)(≥0.7) were used to identify the coexpression of lncRNAs and mRNAs. A* p* < 0.05 indicated statistically significant correlation. In addition, the correlations between lncRNA-TFs and lncRNA-target mRNA-TFs were detected by hypergeometric distribution analysis. The most recent data released by the Encyclopedia of DNA Elements (ENCODE) on TFs and their targets were used in the present analysis. The co expression of lncRNA-mRNA, lncRNA-TFs, and lncRNA-target mRNA-TFs networks were constructed by using Cytoscape software (The Cytoscape Consortium, San Diego, CA, USA).

## 3. Results

### 3.1. Animal Models Construction

**Figure 1:**
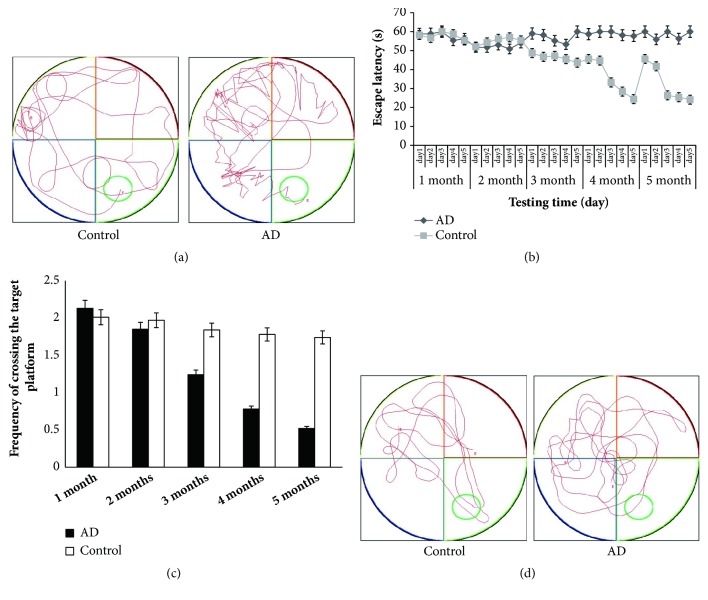
Testing of spatial learning and memory in AD models and controls by Morris water maze. (a) The swim paths and time of finding the hidden platform in the control group was significantly shorter than that of the AD group, indicating that intranasal LPS impairs spatial learning and memory (n = 10/group). (b) Significant difference of escape latency between AD and control groups was observed at the last three days in the fourth and fifth months (*∗* p<0.05) (n = 10/group). (c) Significant difference of the frequency of crossing the target platform between AD and control groups (*∗* p<0.05). Data are expressed as the mean ± standard error of the mean (SEM)(n = 10/group). (d) The image shows the control group had a crossing frequency approximately 2 times the AD group (n = 10/group).

*(1) Behavioral Alterations in AD Models and Controls. *To detect the spatial learning and memory deficits after LPS treatment in the mice, Morris water maze test was performed at the last six days of each month. As shown in Figures 1(a) and 1(b), in visible platform tests mice with chronic intranasal LPS instillation exhibited a longer latency and swimming distances to escape onto the visible platform than that in controls treated with saline, indicating weaker spatial learning ability in AD models than controls. In the probe trial, mice with chronic intranasal LPS instillation spent significantly more time to travel into the fourth quadrant, where the hidden platform was previously placed, than the controls did (Figures 1(c) and 1(d)), which revealed weaker spatial memory ability in the AD model than controls.

**Figure 2:**
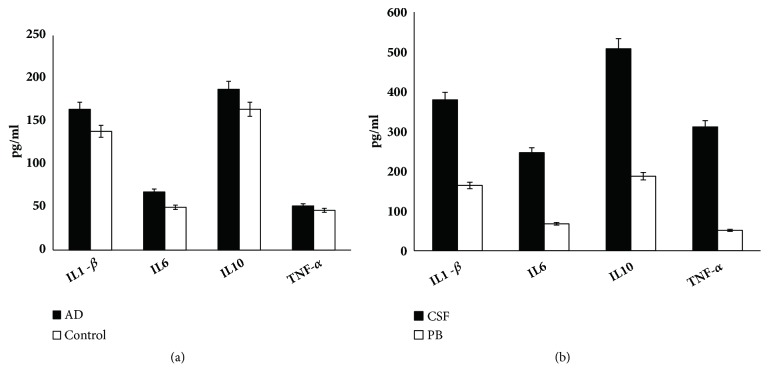
The expression levels of inflammatory factors in AD and control groups. (a) No significant difference was detected for the expression levels of IL6, TNF-*α*, IL1-*β*, and IL10 in peripheral blood of AD and control groups (p>0.05) (n = 6/group). (b) The expression levels of IL6, TNF-*α*, IL1-*β*, and IL10 in cerebrospinal fluid were significantly different from that in peripheral blood of AD group (p>0.05) (n = 6/group). Data are expressed as the mean ± standard error of the mean (SEM).

*(2) Immune Response in Mice with Chronic Intranasal LPS Instillation. *To examine further the influence of intranasal LPS instillation in inducing systemic or locus immune response, we detected the levels of IL1-*β*, IL6, IL10, and TNF-*α* in peripheral blood of AD models and controls, as well as in CSF and PB of AD models. As shown in Figure 2(a), no significant difference in serum IL1-*β*, IL6, IL10, and TNF-*α* between AD models and controls was found. In addition, significant differences were detected for the levels of IL1-*β*, IL6, IL10, and TNF-*α* between CSF and PB in AD models. These results may reveal that the intranasal LPS instillation could only induce locus immune response in central nervous system (CNS), but not systemic immune responses (Figure 2(b)).

**Figure 3:**
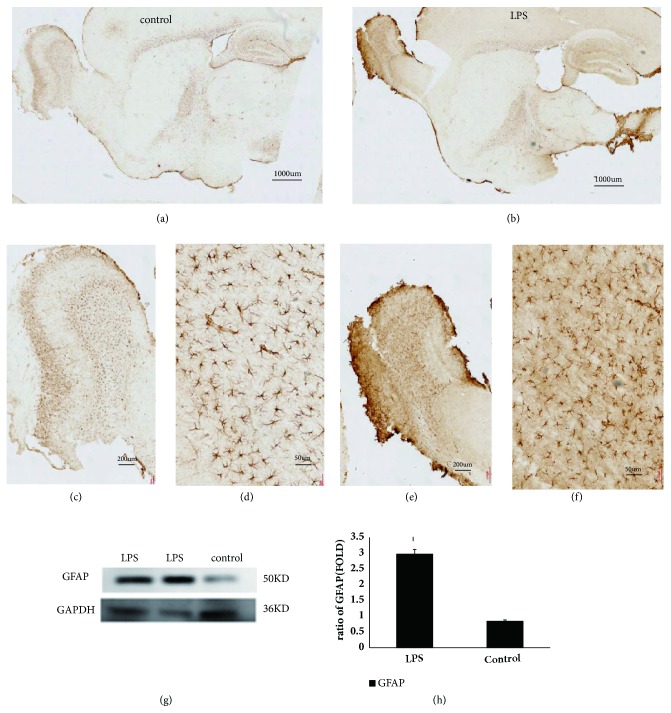
Light microscopic images show the distribution of GFAP immunolabeling across the brain of Intranasal LPS mice and controls. (a)–(f) The images revealed that the GFAP was expressed higher in Intranasal LPS mice than in control mice. And the GFAP expression decreased along the olfactory bulb to the hippocampus. Scale bar= 1000 *μ*m in (a) and (d), 200 *μ*m in (b) and (e), and 50 *μ*m in (c) and (f). (g) Western blot images from Intranasal LPS mice and controls. (h) Quantitative summaries of the protein levels relative to GAPDH as an internal control, expressed as a percentage of GAPDH optical density (o.d.) for the groups (n = 3/group). The ~ 50 kDa GFAP band is not readily seen in control mice compared with Intranasal LPS mice. Statistical results (Kruskal–Wallis nonparametric test with Dunn's multiple post hoc comparison) are shown in the bar graphs, with “*∗*” indicating significant intergroup differences.

*(3) Activation of Astrocyte in AD and Control in Mice. *GFAP is a characteristic marker of astrocytes. When the body is stimulated, the astrocytes in certain parts of the central nervous system appear positive for GFAP. We next defined whether GFAP accumulation was involved in our model by immunohistochemistry and western blots. As shown in Figures 3(a)–3(f), the expression of GFAP in AD models treated with LPS instillation was found to be elevated compared with saline treated controls. Notably, the expression level of GFAP decreased along the olfactory bulb to the hippocampus, which may indicate a potential role of the nasal passage in the pathogenesis of AD. The number of GFAP-positive dots in AD models treated with LPS instillation was significantly increased compared with saline treated controls (Figures 3(g) and 3(h), p<0.05).

### 3.2. Differentially Expressed LncRNAs and MRNAs in Intranasal LPS-Mediated AD Mice

**Figure 4:**
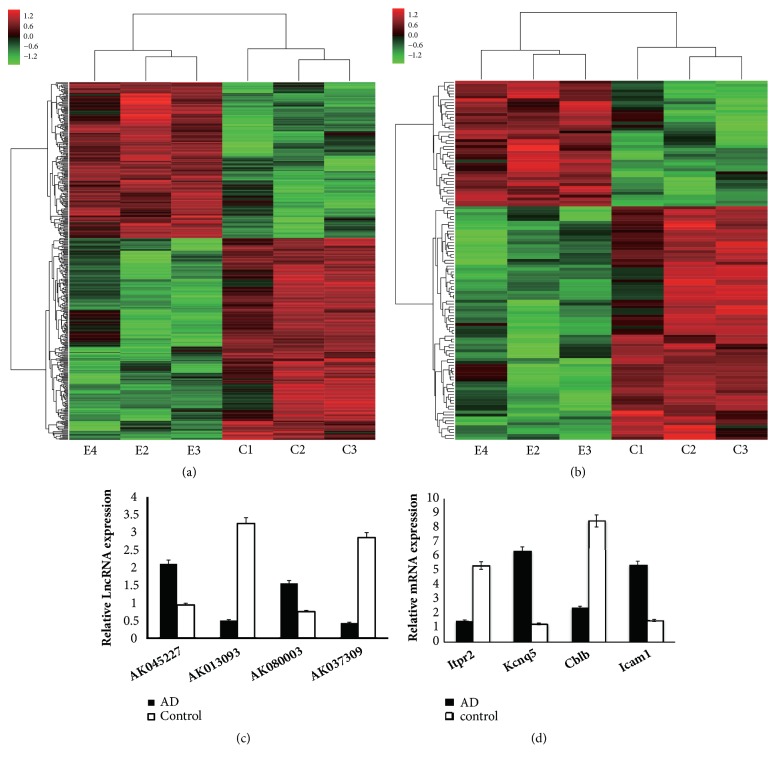
The hierarchical clustering of the differentially expressed lncRNAs (a) and mRNAs (b) in AD(n = 3/group) and control(n = 3/group) hippocampal tissues. (c) and (d) The quantitative real-time PCR (qRT-PCR) validated 4 randomly selected lncRNAs and mRNAs. The qRT-PCR results were consistent with the microarray data. (c) lncRNAS. (d) mRNAs.

The normalized raw data from array image were used to assess the expression levels of lncRNAs and mRNAs in AD mice and controls. A total of 395 significantly dysregulated (172 upregulated and 223 downregulated) lncRNAs were identified. NONMMUT034127.2, with a FC of 9.95, was the most upregulated lncRNA. And, NONMMUT079254.1, with a FC of 7.51, was the most downregulated lncRNA. In addition, 123 significantly dysregulated (43 up-regulated and 80 downregulated) mRNAs were also detected. The most upregulated and downregulated mRNAs were XM_006514426 and NM_146439, with FCs of 9.53 and 4.52 respectively (Figures 4(a) and 4(b), Table s2, and Table s3).

With regard to the AD mouse model conducted by intranasal LPS instillation, 34 mRNAs related to neurons/nervous system diseases, inflammation, and olfactory pathway were selected for further analysis. Among the 34 mRNAs, the most up- and downregulated mRNAs were AK080003 (Cd274) and ENSMUST00000137938 (Itpr2), with FCs of 3.06 and 2.96 separately. A total of 24 lncRNAs coexpressed with the selected 34 mRNAs with the highest Pearson's correlation coefficients were chosen. For the selected 24 lncRNAs, AK158273 (FC=4.89) and NONMMUT079247.1 (FC=2.98) were the most up- and downregulated lncRNAs (Figure s1a and s1b and Table s4). The hierarchical clustering was performed to display the distinguishable lncRNAs and mRNAs expression patterns among AD mice and controls.

The results of microarray were verified by using qRT-PCR. Four lncRNAs and mRNAs were randomly selected and the results were consistent with the microarray chip data (Figures 4(c) and 4(d)).

### 3.3. Coexpression Network and Potential Functions Identification

**Figure 5:**
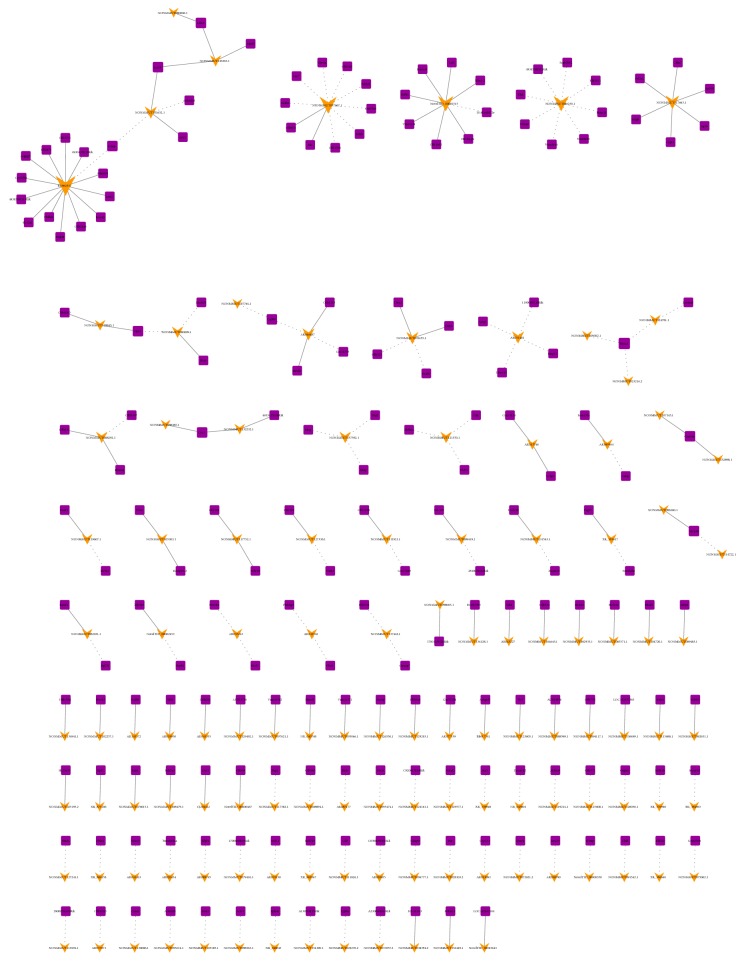
LncRNA-mRNA-network analysis. Purple squares represent dysregulated mRNAs, green arrows represent dysregulated lncRNAs. The dotted lines between lncRNAs and mRNAs indicate a negative correlation, while the solid lines indicate a positive correlation.

The correlation between top100 (100 up- and 100 downregulated) dysregulated lncRNAs and mRNAs were predicted. The* p* value of each lncRNA-mRNA correlation was ranked. The coexpression network was conducted with the selected lncRNA-mRNA correlations with the highest Pearson correlation coefficient (Figure 5). A total of 13246 network nodes and 548607 connections (269022 negative and 279585 positive interactions) were involved in the network. Furthermore, the correlation between the selected 34 mRNAs and their coexpressed lncRNAs were predicted. A total of 359 lncRNAs, 5948 connections(3173 negative and 2775 positive interactions) were involved in the network (Figure s2).

### 3.4. KEGG+GO

For function prediction of lncRNAs, the coexpressed mRNAs for each differentiated lncRNAs were first calculated. And then a functional enrichment analysis of this set of coexpressed mRNAs was conducted. The enriched functional terms were used as the predicted functional term of given lncRNA. The coexpressed mRNAs of lncRNAs were identified by calculating Pearson correlation with correlation* P* value <0.05. Then, the hypergeometric cumulative distribution function was used to calculate the enrichment of functional terms in annotation of coexpressed mRNAs.

**Figure 6:**
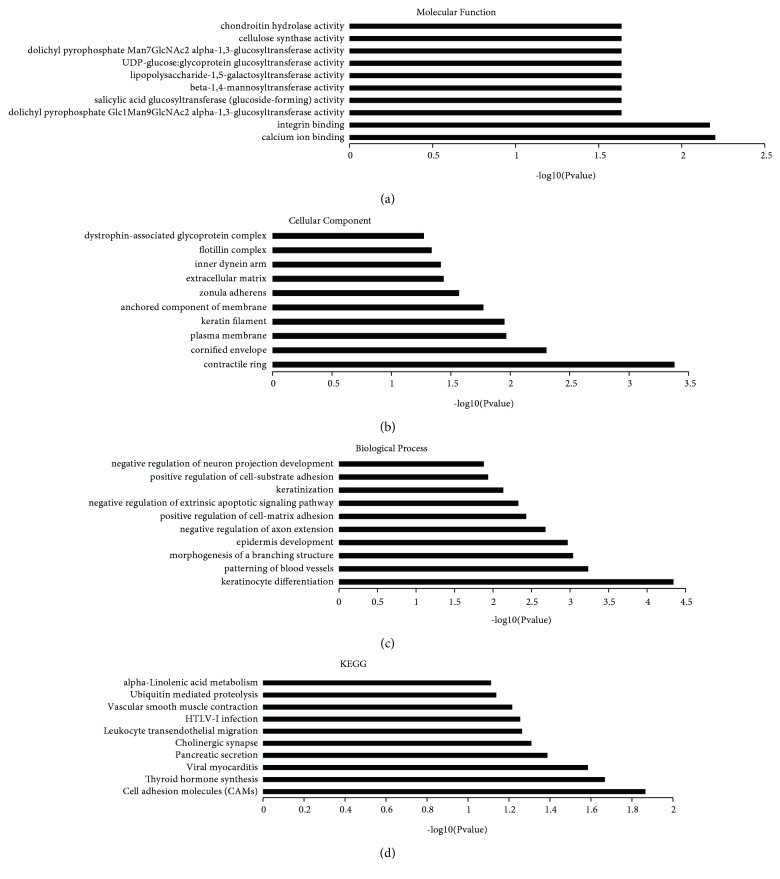
KEGG pathway and GO enrichment analysis of differentially expressed lncRNAs. The top 10 most enriched GO categories and pathways were calculated and plotted. (a) Molecular function; (b) cellular component; (c) biological process; (d) KEGG pathway.

As shown in Figure 6(d) and Table s5, the KEGG analysis indicated that the lncRNA were involved in the inflammation progress (NF-kappa B signaling pathway, TNF signaling pathway, Inflammatory mediator regulation of TRP channels, and cell adhesion molecules (CAMs)) and neuronal function or nervous system diseases (Alzheimer's disease, Huntington's disease, neuroactive ligand-receptor interaction, and synaptic vesicle cycle). Furthermore, the GO enrichment analysis indicated the differentially expressed lncRNAs were mostly enriched in keratinocyte differentiation in its Biological process (Figure 6(c)), contractile ring in its Cellular Component (Figure 6(b)), and calcium ion binding in its molecular function (Figure 6(a)).

### 3.5. LncRNA-TFs Network Analysis

**Figure 7:**
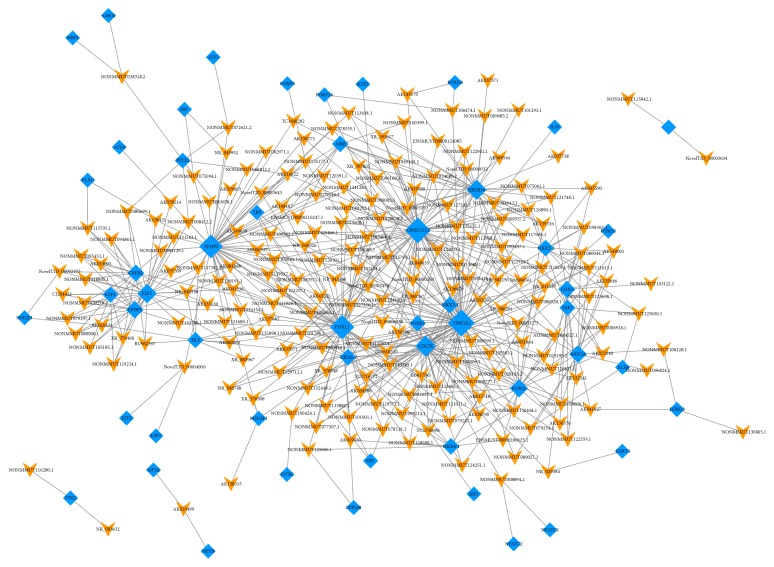
Network of the top 200 most related LncRNA-TFs pairs (the most 5 related lncRNA-TFs pairs according to the P value). Orange arrow: TFs; blue diamonds: lncRNAs.

The TFs were considered to regulate the production of lncRNAs. Thus, the 200 most differentially regulated lncRNAs (100 upregulated and 100 downregulated) were selected and predicted the TFs. These 200 lncRNAs were indicated to be regulated by 248 TFs. The lncRNA-TFs network was conducted on the most 5 related lncRNA-TFs pairs according to the* P* value. The results showed that these lncRNAs were mostly regulated by HMGA2, ONECUT2, FOXO1, CDC5L, TFDP2, ZBTB16, E2F1, NKX3-1, and FOXJ2 (Figure 7). We additionally predicted lncRNA-TFs pairs with the selected 24 lncRNAs related to neuron function/nervous system diseases, inflammation, and olfactory pathway. These 24 lncRNAs were indicated to be regulated by 179 TFs and mostly regulated by TFDP2, ONECUT2, NKX3-1, FOXL1, CDC5L, and FOXJ2 (Figure s3).

### 3.6. LncRNA-Target-TFs Network Analysis

**Figure 8:**
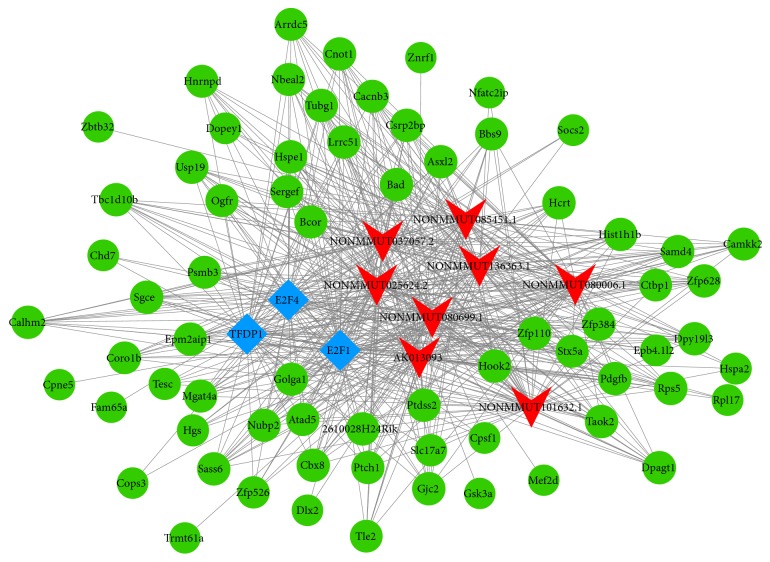
LncRNA-target-TFs network of 20 most differentially expressed lncRNAs. Red arrow: lncRNAs; green round: target mRNAs; blue diamond: TFs.

To investigate the functions of each lncRNA in AD, we analyzed the top 20 mostly differentially expressed lncRNAs (10 upregulated and 10 downregulated) between AD mice and controls. A total of 119 TFs and 5698 mRNAs were predicted to regulate or be the target of these lncRNAs. The lncRNA-target-TFs network was conducted on the most 2 related lncRNA-mRNA and lncRNA-TFs pairs according to the* p* value (Figure 8). Among these TFs, E2F1, E2F4, and TFDP1 were predicted to regulate several lncRNAs. For example, E2F1 and E2F4 were predicted to regulate AK013093, NONMMUT136363.1, NONMMUT101632.1, NONMMUT085451.1, NONMMUT080699.1, NONMMUT080006.1, NONMMUT037057.2, and NONMMUT025624.2. TFDP1 was predicted to regulate AK013093, NONMMUT080699.1, NONMMUT101632.1, and NONMMUT136363.1.

In addition, we analyzed the selected 24 lncRNAs. A total of 183 TFs and 6652 mRNAs were predicted to regulate or be the target of these lncRNAs (Figure s4). Among these TFs, FOXL1, CDC5L, ONECUT2, and CDX1 were predicted to regulate several lncRNAs. CDC5L was predicted to regulate AK045227, AK045590, AK149638, and AK158273. CDX1 was predicted to regulate NONMMUT135177.1, AK045227, and NONMMUT104720.1. ONECUT2 was predicted to regulate NONMMUT099169.1, NONMMUT102111.1, and NONMMUT115705.1. Moreover, most of these TFs were related with inflammation or apoptosis processes.

## 4. Discussion

In the present study, we investigated the expression profiles of lncRNAs in intranasal LPS-mediated Alzheimer's disease model in mice. A total of 395 lncRNAs (172 upregulated and 223 downregulated) and 123 mRNAs (43 upregulated and 80 downregulated) were identified to be differently expressed between AD mice and control. The results of microarray were identified by qRT-PCR.

Dysregulated expression of lncRNAs has been detected in postmortem human brains with AD or late-onset AD [23, 24]. In addition, the expression profiles of lncRNAs in AD models including triple transgenic mice and A*β* intracerebral injection rats were also reported [25–27]. The transgenic model studies have mainly focused on several special genes including APP, PS1/2 [28, 29], and Tau [30]. However, none of these genes can replicate the pathogenesis affected by multiple factors in AD. The A*β* infusion model strongly complements the use of animal models in exploring the role of inflammatory and immune response in the development of AD. However, the A*β* could not explain all aspects of AD pathogenesis [31], especially for the chronic and long-term endotoxin exposure such as LPS. LPS has been used in building AD models by intraperitoneal injection or brain stereotaxic injection [32, 33]. The former might result in systemic inflammation and the latter might simulate acute or subacute response in models. Neither model could replicate the chronic and local inflammatory and progressive degeneration in AD. In addition, LPS was a component of the air pollutants PM2.5 [34], which can be absorbed via the nose and bypass the blood brain barrier. Research has shown that patients with AD and PD have smell loss and olfactory bulb pathology after being exposed in LPS in long terms [35, 36]. In present study, we successfully generated an AD model via unilateral intranasal instillation of LPS, which provided a tool in investigating the intracephalic chronic inflammation-mediated pathogenesis in AD. To our knowledge, this is the first study to investigate the expression profiles of lncRNAs in the hippocampus in LPS-mediated AD model, which may facilitate the understanding of lncRNAs related to the intracephalic chronic inflammation-mediated pathogenesis in AD.

Neuroinflammation induced by A*β*, Tau, and microglia activity in AD has been widely reported [37, 38]. Genes involved in pathways associated with neuroinflammatory have also been identified. According to the KEGG pathway analyses, genes associated with dysregulated lncRNAs in AD models are involved in several inflammation-related pathways such as NF-kappa B signaling pathway, TNF signaling pathway, Jak-STAT signaling pathway, and MAPK signaling pathway. In addition, genes associated with dysregulated lncRNAs in AD models are also involved in synaptic vesicle cycle, cholinergic synapse, dopaminergic synapse, and neuroactive ligand-receptor interaction, which were shown to play an important role in neuronal function and the pathogenesis of neurodegenerative diseases including AD and PD [39, 40]. GO analyzes revealed that these lncRNAs were involved in inflammatory-related biological processes (regulation of p38MAPK cascade, interleukin-1 beta production, and acute inflammatory response to antigenic stimulus) and neuron-related biological processes (negative regulation of axon extension, negative regulation of neuron projection development, and negative regulation of neuron maturation), which are important in learning and memory [41], as well as the development of AD. Compared with the previous study on the expression profiles of lncRNAs in A*β* intracerebral injection AD rat models, several similar KEGG pathways such as insulin signaling pathway, synaptic vesicle cycle, cell adhesion molecules, and neuroactive ligand-receptor interaction were predicted [26], which may indicate an important role of these signaling pathways in neural system, as well as in the pathogenesis of AD.

Furthermore, we identified the ENSMUST00000137938 and ENSMUST00000115299 in the cholinergic synapse pathway. A line of evidence has suggested that the selective loss of cholinergic neurons and decreased synthesis and release of acetyl choline (ACH) were the most important cause of memory loss, cognitive decline, and dementia in AD [42–44]. The lncRNA ENSMUST00000137938 is significantly associated with abundant pathways including apoptosis, long-term potentiation, serotonergic synapse, and dopaminergic synapse, which are shown to be associated with memory and cognition, as well as the development of AD. In addition, ENSMUST00000115299 was found to be only significantly associated with the cholinergic synapse pathway, which may reveal a key role of this lncRNA in the pathogenesis of AD. To confirm this hypothesis, functional identification of ENSMUST00000115299 in AD is necessary in the future.

The lncRNA-TFs network was predicted via hypergeometric distribution analysis. The mostly correlated TFs with top 100 lncRNAs were HMGA2, ONECUT2, FOXO1, CDC5L, TFDP2, ZBTB16, E2F1, NKX3-1, and FOXJ2. Moreover, the TFDP2, ONECUT2, NKX3-1, FOXL1, CDC5L, and FOXJ2 were the mostly related TFs that regulated the production of the 24 selected lncRNAs. Previous studies have shown that TFDP2, NKX3-1, CDC5L, and FOXJ2 were associated with diseases through inducing cell apoptosis, cell cycle regulation, and inflammation [45–47]. Interestingly, HMGA2 was previously focused on enhancing the expression of proinflammatory cytokines including TNF-*α*, IL-6, and IL-1*β*, which is also one of the most important pathogenic mechanisms in AD. However, it is necessary to investigate further how it acts in AD.

We also analyzed the relationship between the top 20 mostly dysregulated lncRNAs with TFs and their mRNAs and found the most likely TFs regulating these lncRNAs were E2F1, E2F4, and TFDP1. Further analysis indicated the AK013093, NONMMUT136363.1, NONMMUT101632.1, NONMMUT085451.1, NONMMUT080699.1, NONMMUT080006.1, NONMMUT037057.2, and NONMMUT025624.2 were predicted to be regulated by both E2F1 and E2F4. As for the selected 24 lncRNAs, the most likely TFs regulating these lncRNAs were FOXL1, CDC5L, ONECUT2, and CDX1. And the AK045227, NONMMUT104720.1, and NONMMUT135177.1 were predicted to be regulated by both CDC5L and CDX1. Notably, the lncRNAs were mostly related to inflammatory and apoptosis process. And the TFs (E2F1, E2F4, CDC5L, and CDX1) were also shown to have function on cell apoptosis [48, 49]. Thus, we presumed that these lncRNAs together with the 4 TFs might affect inflammatory and apoptosis processes and then the pathogenesis of AD.

## 5. Conclusion

We have identified a number of dysregulated lncRNAs and mRNAs that might be potential biomarkers or targets referring to AD. Further investigation is needed to elucidate the detailed mechanisms underlying the regulation of differently expressed lncRNAs.

## Data Availability

The data used to support the findings of this study are included within the article.

## Additional Points

*Highlights.* (1) An intranasal LPS-mediated Alzheimer's disease model in mice was firstly conducted. (2) The expression profiles of lncRNAs in hippocampal showed that 395 lncRNAs and 123 mRNAs were detected to be significantly differently expressed between AD models and controls. (3) The significantly differently expressed mRNAs that correlated with lncRNAs were involved in inflammation, apoptosis, and nervous system related pathways.

## Supplementary Materials

Supplementary Materials Supplementary Figure 1 (Figure s1). The hierarchical clustering of the 34 mRNAs related to neurons/nervous system diseases, inflammation, and olfactory pathway and the 24 lncRNAs coexpressed with the 34 mRNAs with the highest Pearson's correlation coefficients in AD and control hippocampal tissues. a. lncRNAs. b. mRNAs. Supplementary Figure 2 (Figure s2). lncRNA-mRNA-network analysis on 34 selected mRNAs and their coexpressed lncRNAs. Red arrow: lncRNAs; green round: mRNAs. The dotted lines between lncRNAs and mRNAs indicate a negative correlation, while the solid lines indicate a positive correlation. Supplementary Figure 3 (Figure s3) LncRNA-TFs Network of the selected 24 lncRNAs (the most 5 related lncRNA-TFs pairs according to the P value). Orange arrow: lncRNAs; blue diamonds: TFs. Supplementary Figure 4 (Figure s4) lncRNA-target-TFs network of the selected 24 lncRNAs (the most 5 related lncRNA-TFs pairs according to the P value). Orange arrow: lncRNAs; purple square: target mRNAs; Blue diamond: TFs. Supplementary File Table 1 Primers designed for qRT-PCR validation of candidate lncRNAs and mRNAs Supplementary File Table 2. The characters of differently expressed lncRNAs in AD and control. Supplementary File Table 3. The characters of differently expressed mRNAs in AD and control. Supplementary File Table 4. Characters of 34 mRNAs that related to neurons/nervous system diseases, inflammation, and olfactory pathway, and 24 lncRNAs that correlated with 34 mRNAs with the highest Pearson's correlation coefficients. Supplementary File Table 5. The KEGG pathways predicted for differently expressed mRNAs.
