# Supplementary figures and images for: Expression Profiles of Long Noncoding RNAs in Intranasal LPS-Mediated Alzheimer's Disease Model in Mice
Source: Biomed Res Int. 2019 Jan 21;2019:9642589. doi: 10.1155/2019/9642589 (PMC6369469; doi:10.1155/2019/9642589)

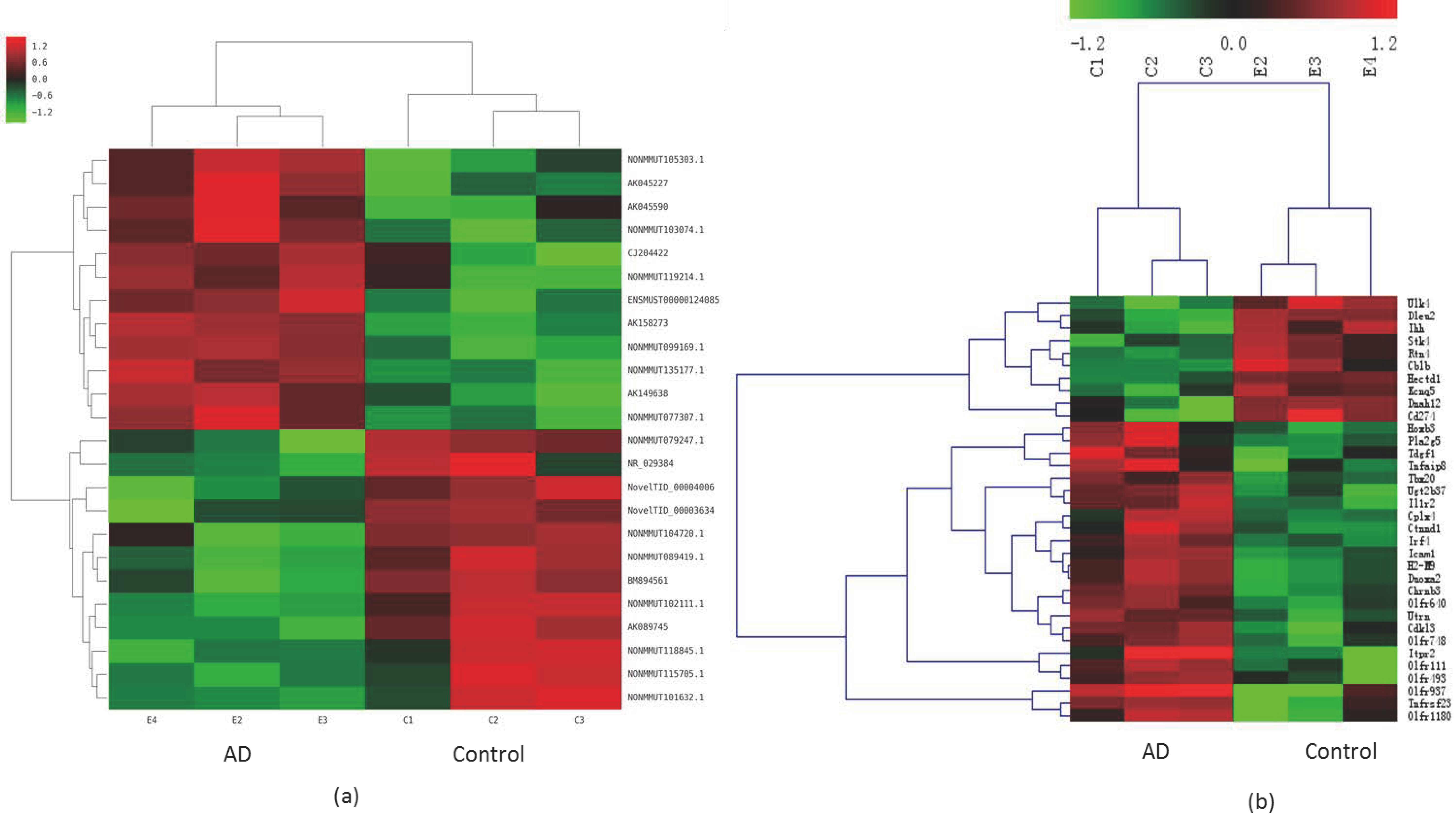

Supplement: Supplementary Materials — Supplementary Figure 1 (Figure s1). The hierarchical clustering of the 34 mRNAs related to neurons/nervous system diseases, inflammation, and olfactory pathway and the 24 lncRNAs coexpressed with the 34 mRNAs with the highest Pearson's correlation coefficients in AD and control hippocampal tissues. a. lncRNAs. b. mRNAs. Supplementary Figure 2 (Figure s2). lncRNA-mRNA-network analysis on 34 selected mRNAs and their coexpressed lncRNAs. Red arrow: lncRNAs; green round: mRNAs. The dotted lines between lncRNAs and mRNAs indicate a negative correlation, while the solid lines indicate a positive correlation. Supplementary Figure 3 (Figure s3) LncRNA-TFs Network of the selected 24 lncRNAs (the most 5 related lncRNA-TFs pairs according to the P value). Orange arrow: lncRNAs; blue diamonds: TFs. Supplementary Figure 4 (Figure s4) lncRNA-target-TFs network of the selected 24 lncRNAs (the most 5 related lncRNA-TFs pairs according to the P value). Orange arrow: lncRNAs; purple square: target mRNAs; Blue diamond: TFs. Supplementary File Table 1 Primers designed for qRT-PCR validation of candidate lncRNAs and mRNAs Supplementary File Table 2. The characters of differently expressed lncRNAs in AD and control. Supplementary File Table 3. The characters of differently expressed mRNAs in AD and control. Supplementary File Table 4. Characters of 34 mRNAs that related to neurons/nervous system diseases, inflammation, and olfactory pathway, and 24 lncRNAs that correlated with 34 mRNAs with the highest Pearson's correlation coefficients. Supplementary File Table 5. The KEGG pathways predicted for differently expressed mRNAs. [file 9642589.f1.zip › Fig s1.tif]
