# Supplementary material for: Expression Profiles of Long Noncoding RNAs in Intranasal LPS-Mediated Alzheimer's Disease Model in Mice
Source: Biomed Res Int. 2019 Jan 21;2019:9642589. doi: 10.1155/2019/9642589 (PMC6369469; doi:10.1155/2019/9642589)
Supplement: Supplementary Materials — Supplementary Figure 1 (Figure s1). The hierarchical clustering of the 34 mRNAs related to neurons/nervous system diseases, inflammation, and olfactory pathway and the 24 lncRNAs coexpressed with the 34 mRNAs with the highest Pearson's correlation coefficients in AD and control hippocampal tissues. a. lncRNAs. b. mRNAs. Supplementary Figure 2 (Figure s2). lncRNA-mRNA-network analysis on 34 selected mRNAs and their coexpressed lncRNAs. Red arrow: lncRNAs; green round: mRNAs. The dotted lines between lncRNAs and mRNAs indicate a negative correlation, while the solid lines indicate a positive correlation. Supplementary Figure 3 (Figure s3) LncRNA-TFs Network of the selected 24 lncRNAs (the most 5 related lncRNA-TFs pairs according to the P value). Orange arrow: lncRNAs; blue diamonds: TFs. Supplementary Figure 4 (Figure s4) lncRNA-target-TFs network of the selected 24 lncRNAs (the most 5 related lncRNA-TFs pairs according to the P value). Orange arrow: lncRNAs; purple square: target mRNAs; Blue diamond: TFs. Supplementary File Table 1 Primers designed for qRT-PCR validation of candidate lncRNAs and mRNAs Supplementary File Table 2. The characters of differently expressed lncRNAs in AD and control. Supplementary File Table 3. The characters of differently expressed mRNAs in AD and control. Supplementary File Table 4. Characters of 34 mRNAs that related to neurons/nervous system diseases, inflammation, and olfactory pathway, and 24 lncRNAs that correlated with 34 mRNAs with the highest Pearson's correlation coefficients. Supplementary File Table 5. The KEGG pathways predicted for differently expressed mRNAs. [file 9642589.f1.zip › Table s2.doc]

Supplementary File Table 2 The characters of differently expressed lncRNAs in AD and control.

| TargetID | GeneSymbol | p | FC | Regulation | C1.txt(normalized) | C2.txt(normalized) | C3.txt(normalized) | E2.txt(normalized) | E3.txt(normalized) | E4.txt(normalized) | chrom | start | end | strand |
| --- | --- | --- | --- | --- | --- | --- | --- | --- | --- | --- | --- | --- | --- | --- |
| NONMMUT098477.1 |  | 0.018359 | 2.125149 | up | 3.85821 | 3.384645 | 3.883005 | 5.049226 | 5.005739 | 4.333587 | chr16 | 40630160 | 40630219 | + |
| NONMMUT023216.2 |  | 0.026497 | 2.0739 | down | 3.549196 | 4.074649 | 3.896709 | 2.416686 | 2.645711 | 3.30112 | chr15 | 37262192 | 37262133 | - |
| NONMMUT119191.1 |  | 0.013394 | 2.940368 | up | 3.085325 | 2.283871 | 2.192796 | 3.621946 | 4.404422 | 4.203613 | chr5 | 1.13E+08 | 1.13E+08 | + |
| NONMMUT100399.1 |  | 4.92E-04 | 2.401501 | up | 3.302833 | 3.027508 | 3.118777 | 4.272746 | 4.584976 | 4.383206 | chr16 | 91138593 | 91138534 | - |
| NONMMUT121011.1 |  | 0.006292 | 2.279712 | up | 2.285893 | 2.917362 | 2.417868 | 3.891514 | 3.799985 | 3.496179 | chr5 | 1.09E+08 | 1.09E+08 | - |
| NONMMUT084642.1 |  | 0.007925 | 2.24575 | up | 2.783347 | 3.339711 | 2.709079 | 4.034971 | 3.935753 | 4.363005 | chr11 | 1.17E+08 | 1.17E+08 | + |
| AK020655 | 9530085P06Rik | 0.039973 | 2.012038 | up | 5.369098 | 4.540948 | 4.472443 | 5.647595 | 6.149045 | 5.61182 | chr8 | 1.24E+08 | 1.24E+08 | - |
| NONMMUT105466.1 |  | 0.012916 | 2.239359 | up | 4.277051 | 3.515703 | 3.814951 | 5.348487 | 4.862238 | 4.886237 | chr19 | 37430121 | 37430180 | + |
| AK053300 |  | 0.016993 | 2.396087 | up | 3.073917 | 2.857834 | 3.741821 | 4.675541 | 4.650733 | 4.129338 | chr9 | 16238040 | 16237981 | - |
| NONMMUT111613.1 |  | 0.004979 | 2.427979 | up | 2.683781 | 3.186015 | 3.005729 | 4.352137 | 3.894979 | 4.467678 | chr3 | 93112872 | 93112931 | + |
| NovelTID_00000032 |  | 0.014273 | 2.155338 | up | 3.528513 | 3.428467 | 3.548393 | 5.115566 | 4.490473 | 4.223076 | chr1 | 59449694 | 59449753 | + |
| NONMMUT059183.2 |  | 0.033304 | 2.017637 | up | 3.460846 | 2.63893 | 2.855722 | 3.59648 | 4.222685 | 4.174333 | chr6 | 1.35E+08 | 1.35E+08 | + |
| AK034598 |  | 0.015621 | 2.264989 | up | 2.636181 | 3.290282 | 2.843282 | 3.991565 | 4.525562 | 3.791129 | chr11 | 1.1E+08 | 1.1E+08 | + |
| XR_376460 | LOC102638939 | 0.034494 | 2.689732 | down | 3.649759 | 5.126864 | 4.662145 | 2.949917 | 3.296984 | 2.909479 | chr4 | 98981892 | 98981951 | + |
| AK040964 |  | 0.028106 | 2.027584 | down | 3.536014 | 3.574555 | 4.249557 | 3.001696 | 2.379911 | 2.919235 | chr6 | 69189315 | 69189256 | - |
| NONMMUT125795.1 |  | 0.042311 | 2.639155 | up | 2.731451 | 2.814303 | 1.452696 | 4.087118 | 3.60778 | 3.50378 | chr7 | 1.03E+08 | 1.03E+08 | + |
| NONMMUT025195.2 |  | 0.024557 | 2.642618 | down | 3.635312 | 3.71943 | 3.329252 | 2.176337 | 1.491462 | 2.81029 | chr16 | 3579169 | 3579110 | - |
| XR_884158 | Gm36368 | 0.002349 | 2.012199 | up | 3.881884 | 3.793255 | 3.919606 | 4.944982 | 5.075999 | 4.600083 | chr18 | 62734257 | 62734198 | - |
| NONMMUT024086.2 |  | 0.009632 | 2.300478 | down | 4.120354 | 3.999849 | 4.339636 | 2.479319 | 3.129951 | 3.24477 | chr15 | 77200923 | 77200864 | - |
| NONMMUT134128.1 |  | 0.014025 | 2.472276 | down | 4.142919 | 5.00386 | 4.727545 | 3.677794 | 3.073917 | 3.205094 | chr9 | 41031848 | 41031789 | - |
| NONMMUT084573.1 |  | 0.005229 | 2.186611 | down | 6.179044 | 6.80612 | 6.659874 | 5.54639 | 5.283112 | 5.429447 | chr11 | 1.13E+08 | 1.13E+08 | + |
| NONMMUT086926.1 |  | 0.027392 | 2.176947 | up | 2.396302 | 3.260637 | 3.466438 | 4.167131 | 4.234466 | 4.088699 | chr12 | 46025123 | 46025182 | + |
| NR_003632 | Gm6787 | 0.042576 | 3.068728 | down | 3.531696 | 3.763753 | 3.697228 | 3.005285 | 2.022585 | 1.111885 | chrX | 7682959 | 7683018 | + |
| NovelTID_00003643 |  | 8.82E-04 | 2.719938 | down | 3.928068 | 4.453138 | 4.320288 | 2.79613 | 2.85371 | 2.720933 | chr19 | 48009923 | 48009982 | + |
| NR_045801 | 5730460C07Rik | 0.028162 | 2.045546 | down | 3.991458 | 4.610145 | 4.875153 | 3.153927 | 3.693631 | 3.531739 | chr3 | 1.53E+08 | 1.53E+08 | - |
| XR_378015 | 1700060L04Rik | 0.021338 | 2.06698 | down | 4.448497 | 4.686456 | 5.028477 | 3.432305 | 3.454622 | 4.133929 | chr6 | 1.24E+08 | 1.24E+08 | + |
| XR_385767 | LOC102634093 | 0.02347 | 2.256631 | up | 2.857138 | 2.71753 | 2.743386 | 4.599525 | 3.602074 | 3.638967 | chr17 | 75341994 | 75341935 | - |
| NONMMUT126056.1 |  | 0.018742 | 2.101183 | up | 6.867169 | 7.657215 | 7.485445 | 8.586581 | 8.515917 | 8.120936 | chr7 | 1.26E+08 | 1.26E+08 | + |
| NONMMUT077667.1 |  | 0.045239 | 2.063855 | down | 7.361926 | 8.268381 | 8.346873 | 6.727227 | 6.810587 | 7.303343 | chr1 | 40691774 | 40691715 | - |
| AK145170 | G930009F23Rik | 0.003627 | 4.319154 | down | 4.12083 | 4.184026 | 3.72095 | 1.469834 | 1.715248 | 2.50848 | chr15 | 38384756 | 38384533 | - |
| AK046497 |  | 0.04923 | 2.134589 | down | 5.070124 | 6.198938 | 5.958406 | 4.746395 | 4.28339 | 4.915806 | chr5 | 1.29E+08 | 1.29E+08 | - |
| NovelTID_00004006 |  | 0.01175 | 2.647059 | down | 3.631047 | 3.939161 | 4.274959 | 2.587753 | 2.97086 | 2.073383 | chr2 | 18736094 | 18736043 | - |
| NR_045705 | I730028E13Rik | 0.042703 | 2.088253 | down | 3.757509 | 4.600637 | 4.694395 | 3.199629 | 3.68155 | 2.98447 | chr9 | 60992969 | 60993028 | + |
| NONMMUT122559.1 |  | 0.026645 | 2.754767 | up | 1.351587 | 2.552182 | 2.694408 | 3.636744 | 3.716342 | 3.630881 | chr6 | 85954235 | 85954294 | + |
| NONMMUT044435.2 |  | 0.023915 | 2.068517 | up | 4.92064 | 5.599432 | 5.317953 | 6.667967 | 6.401905 | 5.913942 | chr3 | 1E+08 | 1E+08 | + |
| NONMMUT076455.1 |  | 0.022155 | 2.2183 | down | 6.694835 | 7.59306 | 7.30596 | 5.724715 | 6.318514 | 6.102261 | chr1 | 1.39E+08 | 1.39E+08 | + |
| NONMMUT112958.1 |  | 0.003976 | 2.582869 | up | 2.084889 | 2.420899 | 2.752213 | 3.998194 | 3.565662 | 3.801069 | chr3 | 24744542 | 24744483 | - |
| AK156542 |  | 0.003733 | 2.007176 | up | 3.00366 | 3.495276 | 3.213362 | 4.079846 | 4.365089 | 4.282866 | chr5 | 4888308 | 4888367 | + |
| NONMMUT103122.1 |  | 0.032809 | 2.691999 | up | 1.830995 | 3.017772 | 3.229887 | 4.305087 | 3.972826 | 4.086776 | chr18 | 26106743 | 26106802 | + |
| NONMMUT115187.1 |  | 0.010821 | 2.095457 | down | 4.8186 | 5.101054 | 5.328061 | 4.182025 | 4.2196 | 3.644295 | chr4 | 83517023 | 83517082 | + |
| AK015584 | 4930478E11Rik | 0.00553 | 2.026235 | down | 4.83164 | 5.092414 | 5.330196 | 3.934585 | 4.30471 | 3.958553 | chr15 | 54813853 | 54813794 | - |
| BU962357 |  | 0.038057 | 2.341305 | down | 4.431571 | 5.648277 | 5.569207 | 4.018874 | 3.826203 | 4.122038 | chr4 | 11347559 | 11347500 | - |
| NONMMUT082031.1 |  | 0.006161 | 2.132243 | down | 4.97932 | 5.206358 | 5.345118 | 3.767302 | 4.106388 | 4.37999 | chr10 | 61560850 | 61560791 | - |
| NONMMUT119297.1 |  | 0.012689 | 2.049076 | down | 4.921668 | 5.338215 | 5.467353 | 3.85821 | 4.418778 | 4.345328 | chr5 | 1.19E+08 | 1.19E+08 | + |
| NONMMUT123524.1 |  | 0.026651 | 2.122067 | up | 3.178793 | 4.176316 | 3.93101 | 5.022804 | 4.849625 | 4.670101 | chr6 | 29786020 | 29785961 | - |
| NONMMUT125630.1 |  | 0.046538 | 3.664092 | up | 1.525826 | 1.720285 | 3.371451 | 4.631574 | 4.00794 | 3.598416 | chr7 | 1.01E+08 | 1.01E+08 | + |
| NONMMUT076210.1 |  | 0.028599 | 2.013528 | down | 10.0768 | 10.44134 | 10.44028 | 9.387143 | 9.744412 | 8.797691 | chr1 | 1.23E+08 | 1.23E+08 | + |
| NovelTID_00000467 |  | 0.028045 | 2.01008 | down | 6.738899 | 7.273325 | 7.634241 | 6.355033 | 6.356042 | 5.913632 | chr10 | 18670368 | 18670427 | + |
| NONMMUT086870.1 |  | 8.44E-04 | 2.109161 | down | 3.55727 | 3.657475 | 3.657133 | 2.605058 | 2.325789 | 2.711023 | chr12 | 35571833 | 35571882 | + |
| NovelTID_00001287 |  | 0.036705 | 2.268862 | down | 2.657091 | 3.489691 | 3.63174 | 1.681325 | 2.063426 | 2.487864 | chr11 | 64576041 | 64575982 | - |
| NR_045952 | Gm4719 | 0.015518 | 2.561522 | down | 4.81698 | 4.779969 | 5.187302 | 3.668565 | 4.051001 | 2.993681 | chr17 | 89784274 | 89784333 | + |
| NONMMUT125698.1 |  | 0.012589 | 7.020942 | up | 0.972296 | 1.28805 | 2.802012 | 5.10339 | 3.976066 | 4.417898 | chr7 | 1.02E+08 | 1.02E+08 | + |
| NONMMUT123779.1 |  | 0.011462 | 2.032152 | up | 2.596228 | 3.19931 | 2.891774 | 4.019873 | 3.619828 | 4.116635 | chr6 | 51482316 | 51507659 | - |
| NONMMUT096824.1 |  | 0.023643 | 2.424241 | down | 4.523141 | 4.249929 | 4.041286 | 2.922659 | 3.599387 | 2.459711 | chr15 | 31169948 | 31169889 | - |
| AK139063 | Dleu2 | 0.004302 | 2.149605 | up | 3.851177 | 3.421536 | 3.383577 | 4.886174 | 4.559214 | 4.523117 | chr14 | 62294983 | 62294924 | - |
| NONMMUT117065.1 |  | 5.74E-04 | 3.726239 | up | 1.780297 | 1.74963 | 2.091934 | 3.927232 | 3.928788 | 3.459001 | chr4 | 1.03E+08 | 1.03E+08 | - |
| NONMMUT028103.2 |  | 0.034036 | 2.354457 | down | 4.077642 | 3.603335 | 3.847296 | 2.527468 | 2.017895 | 3.276727 | chr17 | 7771317 | 7771376 | + |
| NONMMUT072621.2 |  | 0.022597 | 2.355682 | down | 3.681775 | 4.331128 | 4.512145 | 2.561374 | 3.361317 | 2.893924 | chrX | 58872087 | 58872028 | - |
| AK006537 | 1700030E10Rik | 0.013617 | 2.913721 | down | 4.358067 | 5.509496 | 4.790719 | 3.553317 | 3.417298 | 3.059078 | chr10 | 67179356 | 67171447 | - |
| NONMMUT038318.2 |  | 0.048091 | 2.379196 | down | 4.278047 | 5.135675 | 5.334316 | 3.47224 | 4.261202 | 3.263172 | chr2 | 85108233 | 85108174 | - |
| XR_875106 | Gm41289 | 0.021291 | 2.01297 | down | 6.584442 | 7.203765 | 6.920583 | 5.973469 | 6.207713 | 5.499631 | chr15 | 32687334 | 32687275 | - |
| NONMMUT126821.1 |  | 0.007506 | 2.597231 | up | 1.91824 | 2.606223 | 2.719203 | 3.816022 | 3.580791 | 3.977778 | chr7 | 56593035 | 56592976 | - |
| NR_126063 | G630064G18Rik | 0.013425 | 2.011219 | down | 5.392261 | 5.954244 | 6.004233 | 5.030537 | 4.567119 | 4.728873 | chr8 | 38048789 | 38048730 | - |
| NONMMUT092424.1 |  | 3.04E-04 | 2.233732 | up | 2.511574 | 2.664596 | 2.738319 | 3.899204 | 3.655852 | 3.8378 | chr14 | 14804056 | 14804115 | + |
| AK041387 |  | 0.037091 | 2.08921 | up | 6.738577 | 7.032691 | 6.851594 | 8.432035 | 8.08064 | 7.299057 | chr1 | 80725621 | 80725562 | - |
| NONMMUT077446.1 |  | 0.007398 | 3.452013 | down | 4.089126 | 4.50955 | 4.203876 | 3.114658 | 2.340199 | 1.985382 | chr1 | 20852976 | 20852917 | - |
| NONMMUT131689.1 |  | 0.028201 | 2.38322 | down | 5.630361 | 6.544489 | 6.071337 | 4.371723 | 5.28199 | 4.833737 | chr8 | 1.18E+08 | 1.18E+08 | - |
| AK164933 |  | 0.0015 | 2.008905 | down | 3.896844 | 4.128 | 4.016446 | 2.800085 | 3.039458 | 3.182519 | chr2 | 84191172 | 84191113 | - |
| AK087458 |  | 0.036529 | 2.108709 | up | 2.950371 | 2.642052 | 3.392465 | 4.296753 | 4.386778 | 3.530436 | chrX | 1.41E+08 | 1.41E+08 | + |
| NONMMUT104645.1 |  | 0.020722 | 2.063421 | down | 4.89194 | 5.702759 | 5.655474 | 4.167054 | 4.47575 | 4.472255 | chr18 | 57874359 | 57874300 | - |
| NONMMUT090819.1 |  | 0.014232 | 2.941213 | down | 4.507756 | 5.227599 | 5.481634 | 3.709716 | 3.047392 | 3.790648 | chr13 | 7266615 | 7263222 | - |
| NONMMUT135551.1 |  | 0.004134 | 2.134557 | up | 2.433243 | 3.05401 | 2.709724 | 3.895733 | 3.844982 | 3.738071 | chr9 | 1.18E+08 | 1.18E+08 | - |
| AK086354 |  | 0.007063 | 2.042824 | up | 3.717017 | 3.396796 | 3.811334 | 4.850247 | 4.812049 | 4.354543 | chr3 | 1E+08 | 1E+08 | - |
| AK089745 |  | 0.002659 | 2.176368 | down | 4.533739 | 5.028386 | 4.825911 | 3.759645 | 3.49669 | 3.765932 | chr10 | 56301461 | 56301520 | + |
| NONMMUT103074.1 |  | 0.018789 | 2.001069 | up | 4.811354 | 4.291621 | 4.890751 | 6.021446 | 5.557816 | 5.416779 | chr18 | 26013010 | 26013069 | + |
| NONMMUT125606.1 |  | 0.045491 | 2.161314 | up | 4.099363 | 4.214352 | 4.378434 | 6.062817 | 5.186904 | 4.778153 | chr7 | 1.01E+08 | 1.01E+08 | + |
| NONMMUT104020.1 |  | 0.021707 | 2.081438 | down | 6.726557 | 6.983406 | 7.335916 | 5.661623 | 5.802356 | 6.409161 | chr18 | 5459458 | 5459399 | - |
| NONMMUT007670.2 |  | 0.007848 | 2.065111 | down | 4.179028 | 4.595048 | 4.691658 | 3.410008 | 3.213829 | 3.703238 | chr10 | 1.2E+08 | 1.2E+08 | + |
| NONMMUT093474.1 |  | 0.037041 | 2.120889 | down | 4.072177 | 4.94351 | 4.446465 | 2.930408 | 3.518756 | 3.75898 | chr14 | 1.01E+08 | 1.01E+08 | + |
| TC1730998 |  | 0.017269 | 2.667145 | up | 2.459492 | 3.493952 | 2.459423 | 4.150193 | 4.07817 | 4.430391 | chr4 | 1.46E+08 | 1.46E+08 | - |
| NR_040530 | Gm10714 | 0.01261 | 2.643478 | up | 3.276466 | 2.206443 | 2.453003 | 4.123437 | 3.992024 | 4.027763 | chr2 | 1.74E+08 | 1.74E+08 | - |
| AK051481 |  | 0.029888 | 2.032699 | up | 4.038852 | 3.413977 | 3.530011 | 5.170403 | 4.465314 | 4.417311 | chr13 | 89027268 | 89027327 | + |
| AK149001 |  | 0.016577 | 2.272193 | up | 3.340757 | 3.492218 | 4.191392 | 5.142256 | 4.750629 | 4.683738 | chr2 | 82022704 | 82022763 | + |
| AK036814 |  | 0.003694 | 2.072246 | up | 3.425563 | 3.321731 | 3.21416 | 4.657551 | 4.359689 | 4.097797 | chr19 | 59972203 | 59972144 | - |
| NONMMUT106482.1 |  | 0.027829 | 2.126353 | up | 3.052119 | 3.285975 | 2.286616 | 3.833718 | 4.187908 | 3.868226 | chr19 | 59943797 | 59943738 | - |
| AK148487 |  | 0.031661 | 2.337092 | up | 2.81029 | 2.816465 | 1.687402 | 3.708898 | 3.705403 | 3.573999 | chr17 | 86563419 | 86563360 | - |
| AK139336 |  | 0.004591 | 2.477229 | up | 2.444968 | 1.889997 | 2.480182 | 3.824696 | 3.504323 | 3.412308 | chr16 | 96809730 | 96809671 | - |
| NONMMUT129492.1 |  | 0.028158 | 2.060645 | down | 6.263281 | 7.218007 | 7.098966 | 5.670991 | 5.851227 | 5.928748 | chr8 | 1.11E+08 | 1.11E+08 | + |
| NONMMUT078535.1 |  | 0.002441 | 2.171426 | up | 3.072503 | 2.521633 | 2.874032 | 3.920452 | 4.006491 | 3.897152 | chr1 | 1.23E+08 | 1.23E+08 | - |
| AK048935 |  | 0.014855 | 2.35952 | up | 3.473841 | 3.414394 | 3.618698 | 5.12697 | 4.936145 | 4.159297 | chr13 | 89029855 | 89029914 | + |
| NONMMUT089419.1 |  | 0.007508 | 2.830551 | down | 3.670686 | 4.428513 | 4.137877 | 2.254401 | 2.525073 | 2.954353 | chr13 | 27514915 | 27514972 | + |
| NONMMUT124161.1 |  | 0.029389 | 2.007425 | up | 4.756302 | 5.107709 | 4.909079 | 6.195752 | 6.233751 | 5.359626 | chr6 | 96025254 | 96025195 | - |
| AK045590 |  | 0.035203 | 2.258814 | up | 2.801085 | 2.88243 | 3.733381 | 4.764059 | 4.018255 | 4.161277 | chr8 | 14397619 | 14397560 | - |
| NR_015587 | 9330111N05Rik | 0.049695 | 2.344451 | up | 2.230316 | 3.292921 | 3.342338 | 4.470042 | 4.401732 | 3.68155 | chr13 | 81218779 | 81218838 | + |
| NONMMUT082971.1 |  | 0.037459 | 2.605927 | down | 4.136038 | 4.323435 | 4.307387 | 2.574357 | 3.752607 | 2.294507 | chr10 | 1.26E+08 | 1.26E+08 | - |
| NONMMUT130006.1 |  | 0.044814 | 2.456581 | down | 3.516558 | 3.173152 | 3.494205 | 2.141306 | 1.322717 | 2.829937 | chr8 | 15148803 | 15148744 | - |
| NONMMUT111829.1 |  | 0.002393 | 2.498548 | down | 4.163377 | 4.262784 | 3.690346 | 2.83767 | 2.570329 | 2.745236 | chr3 | 1.02E+08 | 1.02E+08 | + |
| NONMMUT108717.1 |  | 0.014038 | 2.018184 | up | 2.854122 | 2.475034 | 3.148933 | 3.997859 | 3.969648 | 3.549756 | chr2 | 16287584 | 16287525 | - |
| NONMMUT105303.1 |  | 0.018015 | 2.377657 | up | 2.674537 | 3.083087 | 3.579351 | 4.624202 | 4.467448 | 3.993945 | chr19 | 22289889 | 22289948 | + |
| TC1779162 |  | 0.049114 | 2.281699 | down | 3.534464 | 3.819715 | 4.009762 | 1.87225 | 2.657037 | 3.264329 | chr2 | 1.49E+08 | 1.49E+08 | - |
| NONMMUT099160.1 |  | 0.015991 | 2.040838 | up | 4.58114 | 4.422103 | 4.117667 | 5.836088 | 5.222149 | 5.150155 | chr16 | 88401120 | 88401179 | + |
| NONMMUT083688.1 |  | 0.045088 | 2.073242 | down | 4.132 | 4.006228 | 4.082818 | 2.29457 | 3.38418 | 3.38663 | chr11 | 55896495 | 55896554 | + |
| NovelTID_00003013 |  | 0.028783 | 2.06935 | down | 4.425786 | 4.243509 | 4.498305 | 2.822961 | 3.877708 | 3.319399 | chr17 | 48063762 | 48063703 | - |
| NONMMUT112770.1 |  | 0.002421 | 2.005887 | down | 3.461151 | 3.312917 | 2.984232 | 2.209263 | 2.201948 | 2.334369 | chr3 | 18218507 | 18218448 | - |
| NR_040645 | 8430436N08Rik | 0.032185 | 2.151398 | down | 5.775272 | 6.298091 | 6.664324 | 5.157399 | 4.741177 | 5.523288 | chr4 | 7500423 | 7500482 | + |
| NONMMUT098187.1 |  | 0.003865 | 2.283375 | down | 4.173686 | 4.26754 | 4.490003 | 3.036063 | 2.866939 | 3.454723 | chr16 | 24053672 | 24053731 | + |
| NONMMUT122393.1 |  | 0.004491 | 2.031871 | down | 5.981181 | 6.338396 | 6.011396 | 4.816401 | 5.216337 | 5.229809 | chr6 | 62931107 | 62931166 | + |
| NovelTID_00003634 |  | 0.04066 | 2.530247 | down | 3.688469 | 3.826467 | 3.540185 | 2.764288 | 2.809754 | 1.463244 | chr19 | 41911156 | 41911215 | + |
| NONMMUT093066.1 |  | 0.019737 | 2.170506 | down | 8.060458 | 8.705218 | 8.688704 | 6.988648 | 7.405489 | 7.70615 | chr14 | 61154881 | 61154940 | + |
| XR_382335 | LOC102640574 | 0.006061 | 2.02013 | down | 3.425226 | 3.332502 | 3.914662 | 2.570329 | 2.635408 | 2.423308 | chr13 | 48748223 | 48748164 | - |
| XR_396660 | LOC102633892 | 0.018831 | 2.045706 | up | 4.900762 | 4.947626 | 4.860882 | 6.42024 | 5.897389 | 5.489433 | chr15 | 95556784 | 95556725 | - |
| AK051864 |  | 0.036951 | 4.238685 | down | 4.250655 | 4.392384 | 3.117057 | 0.957189 | 1.723704 | 2.828352 | chr16 | 57752166 | 57752107 | - |
| NONMMUT111043.1 |  | 0.014563 | 2.046462 | down | 3.458028 | 3.348773 | 3.942691 | 2.275389 | 2.865577 | 2.509133 | chr3 | 45406413 | 45420352 | + |
| AK156546 |  | 0.017146 | 3.186514 | down | 3.475592 | 3.166682 | 3.748457 | 2.265754 | 1.015561 | 2.093479 | chr15 | 23655610 | 23655551 | - |
| NovelTID_00005703 |  | 0.016318 | 3.049562 | down | 4.382049 | 4.164585 | 4.195579 | 1.879512 | 2.812706 | 3.224189 | chr7 | 54276656 | 54276715 | + |
| NONMMUT120617.1 |  | 0.019385 | 2.05382 | up | 3.450459 | 3.294658 | 3.884353 | 4.997734 | 4.424849 | 4.321817 | chr5 | 67297641 | 67297582 | - |
| BM894561 |  | 0.009093 | 2.485682 | down | 6.333239 | 6.673628 | 6.417749 | 4.768159 | 5.068039 | 5.647493 | chr2 | 1.56E+08 | 1.56E+08 | - |
| BB617541 |  | 0.030679 | 3.028189 | down | 7.661587 | 8.304104 | 8.147793 | 5.640482 | 6.486344 | 7.19129 | chr8 | 64400862 | 64400803 | - |
| NONMMUT116280.1 |  | 0.025888 | 3.299557 | down | 3.55742 | 3.420408 | 3.52806 | 2.639031 | 1.780766 | 0.919272 | chr4 | 22383730 | 22383671 | - |
| NONMMUT115946.1 |  | 0.02637 | 2.087145 | down | 4.054088 | 3.606653 | 3.187293 | 2.194781 | 2.769831 | 2.698829 | chr4 | 1.44E+08 | 1.44E+08 | + |
| AK053250 |  | 0.035021 | 2.015139 | up | 4.225755 | 3.821714 | 3.979089 | 4.586705 | 5.596577 | 4.875914 | chr6 | 66870338 | 66870397 | + |
| NONMMUT083072.1 |  | 0.018196 | 2.136384 | down | 6.357145 | 6.987271 | 6.72261 | 5.404436 | 5.349604 | 6.027473 | chr11 | 4021916 | 4021975 | + |
| NONMMUT075062.1 |  | 0.005724 | 2.407985 | down | 4.743385 | 5.22345 | 4.771363 | 3.301441 | 3.747317 | 3.885961 | chr1 | 16388220 | 16388279 | + |
| NONMMUT110464.1 |  | 0.00917 | 2.202237 | up | 3.404189 | 3.864397 | 3.642294 | 4.96594 | 4.988533 | 4.373316 | chr3 | 5525545 | 5525604 | + |
| NONMMUT122952.1 |  | 0.010164 | 2.30615 | up | 2.924505 | 2.622991 | 2.990094 | 4.301211 | 4.276354 | 3.576482 | chr6 | 1.2E+08 | 1.2E+08 | + |
| NONMMUT099169.1 |  | 0.001323 | 2.165181 | up | 3.763553 | 3.308404 | 3.513282 | 4.708036 | 4.555047 | 4.665621 | chr16 | 88440151 | 88440210 | + |
| AK019930 | 5330433J24Rik | 0.029073 | 2.370064 | up | 5.274673 | 5.877827 | 5.758471 | 7.482576 | 6.796939 | 6.366231 | chr15 | 50572277 | 50572218 | - |
| NONMMUT097621.1 |  | 0.020826 | 2.092789 | up | 5.909702 | 6.279597 | 6.214088 | 7.628924 | 7.253082 | 6.71766 | chr15 | 95571986 | 95571927 | - |
| NONMMUT085044.1 |  | 0.017007 | 2.027386 | up | 4.031094 | 3.557343 | 3.16738 | 4.473487 | 4.635785 | 4.705408 | chr11 | 32037362 | 32037303 | - |
| NONMMUT135618.1 |  | 0.02996 | 2.123968 | down | 6.195381 | 6.650745 | 6.568073 | 5.008513 | 5.171959 | 5.973443 | chr9 | 1.22E+08 | 1.22E+08 | - |
| NONMMUT103408.1 |  | 0.010091 | 2.386127 | down | 4.191255 | 4.180794 | 4.057814 | 2.348929 | 3.15778 | 3.159142 | chr18 | 42506120 | 42506179 | + |
| NONMMUT076015.1 |  | 0.036445 | 2.055641 | down | 12.10547 | 12.50109 | 12.50109 | 10.91285 | 11.14266 | 11.93339 | chr1 | 95553093 | 95553152 | + |
| NONMMUT126724.1 |  | 0.041315 | 2.007155 | down | 7.233399 | 7.130032 | 7.164558 | 5.741375 | 5.934488 | 6.83667 | chr7 | 47233509 | 47233450 | - |
| XR_382256 | Gm32448 | 0.014189 | 3.312521 | down | 3.876337 | 3.984309 | 3.959824 | 1.403399 | 2.773791 | 2.459492 | chr13 | 41506460 | 41506519 | + |
| AK019580 | 4930421C12Rik | 0.040177 | 3.477384 | down | 4.056669 | 4.121535 | 4.489026 | 1.254512 | 2.977241 | 3.041471 | chr5 | 39833416 | 39833357 | - |
| NONMMUT134190.1 |  | 0.024382 | 2.384729 | up | 4.677175 | 4.077039 | 4.186249 | 6.087846 | 5.58012 | 5.033974 | chr9 | 42321291 | 42321232 | - |
| NONMMUT129577.1 |  | 0.009713 | 2.516501 | down | 4.544232 | 4.209487 | 4.456799 | 2.73487 | 2.878376 | 3.603015 | chr8 | 1.15E+08 | 1.15E+08 | + |
| NONMMUT137442.1 |  | 0.04457 | 2.025734 | down | 9.011316 | 9.596318 | 9.662796 | 8.037437 | 8.211011 | 8.966648 | chrX | 1.39E+08 | 1.39E+08 | - |
| AK132528 |  | 0.020945 | 2.021303 | up | 3.739942 | 3.861244 | 3.960712 | 5.166783 | 5.105195 | 4.335776 | chr12 | 1.09E+08 | 1.09E+08 | - |
| XR_384963 | LOC102631826 | 0.01093 | 2.095332 | down | 6.560006 | 6.794884 | 6.83891 | 5.422804 | 5.462945 | 6.106513 | chr16 | 73099293 | 73099352 | + |
| NONMMUT101796.1 |  | 0.032839 | 2.426303 | up | 3.606704 | 3.355123 | 3.6513 | 4.431392 | 5.593615 | 4.4244 | chr17 | 13224076 | 13224017 | - |
| NONMMUT115688.1 |  | 0.028636 | 2.633965 | down | 4.857067 | 4.892547 | 4.816036 | 2.974113 | 3.112029 | 4.287801 | chr4 | 1.3E+08 | 1.3E+08 | + |
| NONMMUT076514.1 |  | 0.042808 | 2.079989 | down | 5.035256 | 5.137744 | 6.066179 | 4.112157 | 4.327805 | 4.629489 | chr1 | 1.44E+08 | 1.44E+08 | + |
| NONMMUT115882.1 |  | 0.040734 | 2.621583 | down | 6.452243 | 7.087332 | 7.168515 | 5.292755 | 4.941496 | 6.302527 | chr4 | 1.41E+08 | 1.41E+08 | + |
| NONMMUT114722.1 |  | 0.03223 | 2.067011 | down | 7.460984 | 7.758995 | 7.70612 | 6.392997 | 6.183911 | 7.206553 | chr4 | 21596562 | 21596620 | + |
| NovelTID_00003476 |  | 0.037447 | 2.830903 | down | 4.488004 | 5.028319 | 4.779807 | 2.354466 | 3.558807 | 3.879071 | chr18 | 8153164 | 8153105 | - |
| XR_400120 | LOC102633794 | 0.025817 | 2.098678 | down | 5.548833 | 5.958773 | 5.61912 | 4.218577 | 4.5247 | 5.175007 | chr2 | 1.15E+08 | 1.15E+08 | - |
| NONMMUT104720.1 |  | 0.020482 | 2.031233 | down | 6.256126 | 6.316498 | 6.425696 | 4.968181 | 5.119028 | 5.844043 | chr18 | 62312698 | 62312639 | - |
| NONMMUT137392.1 |  | 0.025712 | 2.139353 | down | 3.463162 | 4.226907 | 4.477363 | 2.861491 | 2.885973 | 3.128443 | chrX | 1.3E+08 | 1.3E+08 | - |
| NONMMUT036304.2 |  | 0.03811 | 2.007886 | down | 6.954754 | 7.300079 | 7.357032 | 5.921375 | 5.865829 | 6.807628 | chr2 | 26155124 | 26156123 | + |
| NONMMUT079247.1 |  | 0.022558 | 2.980267 | down | 3.820828 | 3.560491 | 3.354021 | 2.191484 | 1.208749 | 2.608783 | chr1 | 1.69E+08 | 1.69E+08 | - |
| NONMMUT101001.1 |  | 0.042085 | 2.963247 | down | 3.873216 | 4.743527 | 4.867858 | 3.155898 | 2.095598 | 3.531568 | chr17 | 36634424 | 36634483 | + |
| NONMMUT109302.1 |  | 0.031176 | 2.726003 | down | 3.802651 | 4.592727 | 4.401582 | 2.239402 | 2.695925 | 3.521275 | chr2 | 84331787 | 84331728 | - |
| NONMMUT089134.1 |  | 0.021029 | 2.122816 | down | 5.117107 | 5.364834 | 5.513678 | 3.763067 | 4.275292 | 4.699323 | chr12 | 1.14E+08 | 1.14E+08 | - |
| NONMMUT068017.2 |  | 0.022569 | 2.203722 | down | 4.277867 | 3.486183 | 3.841399 | 3.147157 | 2.622058 | 2.416406 | chr9 | 12865839 | 12865898 | + |
| NONMMUT118542.1 |  | 0.018805 | 2.010468 | down | 7.002347 | 7.465399 | 7.507469 | 6.176152 | 6.048632 | 6.727836 | chr5 | 66858007 | 66859058 | + |
| NONMMUT108085.1 |  | 0.013939 | 2.319338 | down | 4.418364 | 4.816787 | 4.654025 | 3.489313 | 2.922196 | 3.836528 | chr2 | 1.47E+08 | 1.47E+08 | + |
| NONMMUT080404.1 |  | 0.045747 | 2.047951 | down | 6.722415 | 6.756618 | 6.856057 | 5.46446 | 5.311606 | 6.456481 | chr10 | 72097078 | 72097137 | + |
| NONMMUT089483.1 |  | 0.013137 | 2.283018 | down | 6.104382 | 6.574823 | 6.564279 | 5.083966 | 4.907881 | 5.678811 | chr13 | 32958777 | 32958836 | + |
| NONMMUT129203.1 |  | 0.025435 | 2.229601 | down | 6.362598 | 7.090679 | 7.199798 | 5.356839 | 5.764813 | 6.061069 | chr8 | 94926104 | 94926163 | + |
| NAP068908-1 |  | 0.038938 | 2.208989 | down | 5.256158 | 5.553541 | 4.986053 | 3.624333 | 3.967973 | 4.773286 | chr2 | 1.3E+08 | 1.3E+08 | + |
| NONMMUT086528.1 |  | 0.016149 | 2.322333 | up | 2.822893 | 3.850232 | 3.142854 | 4.470651 | 4.521832 | 4.470221 | chr12 | 24447851 | 24447910 | + |
| NONMMUT084527.1 |  | 0.024442 | 5.200749 | down | 3.942982 | 4.700085 | 4.441011 | 1.704009 | 1.043326 | 3.200586 | chr11 | 1.09E+08 | 1.09E+08 | + |
| NONMMUT046157.2 |  | 0.025882 | 2.139581 | down | 5.758598 | 5.607637 | 6.141749 | 4.232952 | 4.805514 | 5.177534 | chr4 | 10674089 | 10674030 | - |
| NONMMUT121570.1 |  | 0.022629 | 2.052962 | down | 5.516452 | 5.936307 | 6.052103 | 4.491651 | 4.635546 | 5.264544 | chr5 | 1.42E+08 | 1.42E+08 | - |
| NR_040696 | 4930448I18Rik | 0.001844 | 2.191358 | down | 5.827014 | 5.966596 | 6.045079 | 4.903972 | 4.538712 | 5.000529 | chr5 | 50547219 | 50547278 | + |
| NONMMUT108479.1 |  | 0.046573 | 2.122297 | down | 12.16155 | 12.5066 | 12.54308 | 10.94181 | 10.97155 | 12.04099 | chr2 | 1.72E+08 | 1.72E+08 | + |
| NovelTID_00000350 |  | 0.035484 | 4.325893 | down | 5.861944 | 6.219377 | 6.355946 | 3.033186 | 3.783911 | 5.281176 | chr1 | 1.57E+08 | 1.57E+08 | - |
| NONMMUT088599.1 |  | 0.031993 | 2.33884 | down | 5.176911 | 5.295305 | 5.266564 | 3.581239 | 3.707435 | 4.772727 | chr12 | 76843101 | 76843042 | - |
| NovelTID_00006350 |  | 0.0177 | 2.531895 | down | 4.068849 | 4.337934 | 4.535687 | 2.518876 | 2.819386 | 3.583556 | chr9 | 86900893 | 86900952 | + |
| NONMMUT096777.1 |  | 0.024273 | 2.410561 | down | 3.613852 | 3.408505 | 3.519262 | 2.084085 | 1.725979 | 2.92345 | chr15 | 26201387 | 26201328 | - |
| TC1618330 |  | 0.026233 | 3.081677 | up | 2.768165 | 2.985456 | 2.049756 | 4.89586 | 4.189154 | 3.589511 | chr5 | 1.23E+08 | 1.23E+08 | - |
| NR_108059 | 4930463O16Rik | 0.011591 | 2.081281 | down | 7.826716 | 8.45243 | 8.296781 | 6.83731 | 7.276096 | 7.290106 | chr10 | 83956560 | 83956619 | + |
| NONMMUT098363.1 |  | 0.031266 | 2.378533 | up | 2.48727 | 2.941806 | 3.614556 | 4.666273 | 4.050722 | 4.076854 | chr16 | 40025444 | 40025503 | + |
| NONMMUT009557.2 |  | 0.016757 | 2.558561 | down | 3.642501 | 3.512521 | 3.67834 | 2.036233 | 1.809999 | 2.921133 | chr11 | 49218974 | 49219033 | + |
| NONMMUT092631.1 |  | 0.005366 | 2.190753 | up | 2.724481 | 3.185508 | 2.972396 | 4.385325 | 4.044615 | 3.846726 | chr14 | 30387254 | 30387313 | + |
| NONMMUT117382.1 |  | 0.018693 | 2.340498 | down | 6.783743 | 7.791084 | 7.564229 | 5.962797 | 6.295242 | 6.200571 | chr4 | 1.32E+08 | 1.32E+08 | - |
| NONMMUT094117.1 |  | 0.042905 | 2.026513 | down | 8.283404 | 9.058798 | 9.276768 | 7.741029 | 7.625464 | 8.195477 | chr14 | 26148870 | 26148811 | - |
| NONMMUT132232.1 |  | 0.001917 | 2.835288 | down | 4.236286 | 4.308851 | 4.477298 | 2.723246 | 2.572178 | 3.216527 | chr9 | 30297482 | 30297541 | + |
| XR_376908 | LOC102637363 | 0.025849 | 8.2433 | up | 3.120191 | 0.942409 | 1.614719 | 5.471301 | 5.597276 | 3.738407 | chr5 | 38705856 | 38705913 | + |
| NR_045469 | 4930455J16Rik | 0.017387 | 2.770593 | down | 6.525247 | 6.632138 | 6.703977 | 4.511917 | 5.13724 | 5.801621 | chr13 | 58845304 | 58845245 | - |
| NONMMUT091542.1 |  | 0.009824 | 2.285307 | down | 5.791285 | 6.460692 | 6.538998 | 5.081995 | 4.89214 | 5.239676 | chr13 | 59532722 | 59532663 | - |
| NONMMUT129712.1 |  | 0.043405 | 2.427245 | down | 9.505749 | 10.20897 | 10.15298 | 8.117173 | 8.52055 | 9.392016 | chr8 | 1.26E+08 | 1.26E+08 | + |
| AK037071 |  | 0.034597 | 2.701848 | down | 8.948394 | 9.901391 | 10.00018 | 7.792346 | 7.964352 | 8.791426 | chr17 | 30237343 | 30237402 | + |
| NONMMUT088292.1 |  | 0.035338 | 2.634565 | down | 4.620043 | 4.839789 | 5.086711 | 2.826902 | 3.260548 | 4.266398 | chr12 | 36169865 | 36169806 | - |
| AK087177 |  | 0.029889 | 2.001932 | down | 7.646686 | 8.485293 | 8.301391 | 6.831843 | 7.20352 | 7.393824 | chr17 | 31650490 | 31650549 | + |
| NONMMUT132898.1 |  | 0.015303 | 2.48892 | up | 3.595721 | 2.508305 | 3.062701 | 4.414328 | 4.481203 | 4.217757 | chr9 | 80002521 | 80002580 | + |
| NONMMUT064175.2 |  | 0.037999 | 2.019498 | up | 2.94836 | 2.298846 | 3.211151 | 3.664878 | 4.217007 | 3.618464 | chr7 | 1.48E+08 | 1.48E+08 | - |
| NONMMUT129212.1 |  | 0.034499 | 2.021378 | up | 3.919143 | 3.729361 | 2.975487 | 4.744501 | 4.652499 | 4.273007 | chr8 | 94993409 | 94993468 | + |
| NONMMUT127336.1 |  | 0.048242 | 2.654189 | down | 4.926008 | 5.592647 | 5.334398 | 3.355237 | 3.476016 | 4.796985 | chr7 | 82908657 | 82908598 | - |
| NONMMUT122041.1 |  | 0.017539 | 2.140461 | up | 2.375266 | 2.969883 | 3.285619 | 4.130666 | 3.973484 | 3.820384 | chr6 | 37122127 | 37122186 | + |
| NONMMUT093664.1 |  | 0.005638 | 2.093085 | up | 4.379132 | 4.888135 | 4.743223 | 5.987037 | 5.600735 | 5.619612 | chr14 | 1.12E+08 | 1.12E+08 | + |
| AK149638 |  | 0.010476 | 2.798899 | up | 3.127144 | 2.614954 | 2.163259 | 4.341565 | 3.783042 | 4.23533 | chr4 | 60549203 | 60549262 | + |
| XR_378948 | LOC102634125 | 0.011135 | 2.80238 | down | 6.386581 | 7.150673 | 7.02433 | 4.99451 | 5.30667 | 5.800447 | chr8 | 48831698 | 48831639 | - |
| AK136879 |  | 0.003635 | 3.807037 | up | 2.786568 | 2.404505 | 2.228259 | 4.01852 | 4.921149 | 4.265667 | chr2 | 91038944 | 91038885 | - |
| NONMMUT129587.1 |  | 0.026646 | 2.070037 | down | 4.502419 | 5.281642 | 5.451371 | 4.213478 | 3.93045 | 3.942534 | chr8 | 1.15E+08 | 1.15E+08 | + |
| NONMMUT100626.1 |  | 0.018405 | 2.665153 | down | 7.748586 | 8.552016 | 8.573051 | 6.639623 | 6.617587 | 7.373789 | chr17 | 14334300 | 14334359 | + |
| NONMMUT095214.1 |  | 0.024199 | 2.534505 | up | 2.979976 | 3.695529 | 4.152955 | 5.097584 | 5.137696 | 4.618294 | chr15 | 8433292 | 8433351 | + |
| NONMMUT105465.1 |  | 0.024327 | 2.011216 | up | 2.752138 | 3.378686 | 2.41164 | 3.833434 | 3.801139 | 3.932094 | chr19 | 37425713 | 37425772 | + |
| NONMMUT106228.1 |  | 0.039849 | 2.815509 | down | 6.174377 | 5.38792 | 5.131013 | 4.63453 | 4.246005 | 3.332587 | chr19 | 44311166 | 44311107 | - |
| NONMMUT108606.1 |  | 0.005285 | 2.325401 | up | 2.556559 | 3.098375 | 3.108166 | 4.248033 | 3.889597 | 4.277907 | chr2 | 1.81E+08 | 1.81E+08 | + |
| NONMMUT077307.1 |  | 0.005381 | 2.440464 | up | 2.713835 | 2.899716 | 2.399381 | 4.303265 | 3.676419 | 3.894714 | chr1 | 9511950 | 9511891 | - |
| NONMMUT087499.1 |  | 0.021037 | 2.113029 | down | 5.784842 | 6.491821 | 6.765779 | 5.295788 | 5.247783 | 5.260935 | chr12 | 91637076 | 91637135 | + |
| AK021040 | Cdh10 | 0.007426 | 2.1695 | up | 2.639458 | 3.218434 | 3.259153 | 4.06431 | 4.352683 | 4.052139 | chr15 | 18859936 | 18859995 | + |
| NONMMUT137024.1 |  | 0.044112 | 2.221577 | down | 4.921997 | 4.987132 | 5.121502 | 3.662009 | 3.298396 | 4.615474 | chrX | 71182502 | 71182443 | - |
| AK157082 |  | 0.026162 | 3.857981 | up | 4.628861 | 3.217763 | 3.310235 | 6.020244 | 5.982022 | 4.998131 | chr8 | 93915133 | 93915074 | - |
| XR_387492 | Gm9530 | 0.021505 | 2.029573 | down | 4.134535 | 4.940968 | 4.481203 | 3.531568 | 3.219028 | 3.742579 | chr1 | 1.57E+08 | 1.57E+08 | - |
| NONMMUT115594.1 |  | 0.015818 | 4.937103 | down | 3.633121 | 4.695972 | 4.697316 | 1.144014 | 2.565575 | 2.405825 | chr4 | 1.26E+08 | 1.26E+08 | + |
| ENSMUST00000210247.1 |  | 0.007241 | 2.165758 | up | 3.183064 | 2.687506 | 2.678927 | 4.146419 | 4.069449 | 3.678242 | chr7 | 1.14E+08 | 1.14E+08 | + |
| NONMMUT102111.1 |  | 0.005913 | 2.7791 | down | 6.976936 | 7.766426 | 7.763729 | 5.890869 | 6.010176 | 6.182192 | chr17 | 43267485 | 43267426 | - |
| NONMMUT085451.1 |  | 0.036573 | 2.560137 | down | 4.925762 | 6.073816 | 6.344661 | 4.450903 | 4.512145 | 4.312528 | chr11 | 72039237 | 72039178 | - |
| NONMMUT107145.1 |  | 0.016809 | 2.129076 | up | 3.192194 | 2.282965 | 2.718584 | 3.856825 | 3.947794 | 3.659806 | chr2 | 62919757 | 62919816 | + |
| XR_381562 | LOC102636270 | 0.001154 | 2.183501 | down | 4.429618 | 4.739721 | 4.738019 | 3.508552 | 3.356201 | 3.662675 | chr12 | 44728228 | 44728169 | - |
| AK081868 |  | 0.00644 | 3.120126 | down | 6.660288 | 7.323863 | 7.614759 | 5.438356 | 5.834533 | 5.401207 | chr4 | 1.15E+08 | 1.15E+08 | + |
| AK041027 | Ptgr2 | 0.028982 | 2.821945 | down | 7.83839 | 7.246975 | 6.40864 | 5.72209 | 5.934273 | 5.347575 | chr12 | 85628790 | 85628849 | + |
| NONMMUT126957.1 |  | 0.031222 | 2.081389 | up | 3.442641 | 2.63654 | 3.622328 | 4.059474 | 4.43307 | 4.381603 | chr7 | 67523523 | 67523464 | - |
| TC1716772 |  | 0.008949 | 2.155907 | up | 4.727692 | 4.807961 | 5.042246 | 6.256763 | 6.094432 | 5.551589 | chr7 | 1.42E+08 | 1.42E+08 | - |
| XR_397005 | LOC102640069 | 0.031786 | 3.205664 | down | 9.666864 | 10.18304 | 10.21254 | 7.883153 | 7.821579 | 9.315846 | chr16 | 37978909 | 37978850 | - |
| AK015804 | 4930516E23Rik | 0.043666 | 2.057594 | up | 2.525725 | 3.504276 | 3.263482 | 4.502974 | 3.800596 | 4.112789 | chr10 | 61054890 | 61054949 | + |
| NONMMUT124781.1 |  | 0.026189 | 2.134167 | down | 9.965788 | 10.39823 | 10.34551 | 8.693427 | 9.058798 | 9.67629 | chr7 | 20402447 | 20402506 | + |
| NONMMUT136363.1 |  | 0.041591 | 2.131505 | down | 4.980215 | 6.076353 | 6.090344 | 4.634388 | 4.566661 | 4.670247 | chrX | 1.31E+08 | 1.31E+08 | + |
| NONMMUT121748.1 |  | 0.033692 | 3.459141 | up | 2.665536 | 1.184071 | 2.746682 | 3.631914 | 4.458223 | 3.877391 | chr6 | 5409429 | 5409488 | + |
| NR_046026 | Gm15713 | 0.021484 | 2.880848 | up | 2.949869 | 2.681699 | 1.925805 | 4.597963 | 3.867428 | 3.671461 | chr16 | 43410767 | 43410708 | - |
| NONMMUT050159.2 |  | 0.001349 | 2.097663 | down | 10.13827 | 10.18424 | 9.825311 | 8.985408 | 9.104188 | 8.851872 | chr4 | 1.37E+08 | 1.37E+08 | + |
| NONMMUT127241.1 |  | 0.007957 | 3.688262 | down | 4.69667 | 4.82626 | 4.896853 | 2.660751 | 2.440178 | 3.670032 | chr7 | 76593746 | 76593687 | - |
| NR_073462 | 5730488B01Rik | 0.026059 | 2.276101 | down | 6.412002 | 7.241393 | 7.552779 | 5.910436 | 5.949302 | 5.786742 | chr4 | 44717923 | 44717982 | + |
| NONMMUT078519.1 |  | 0.027998 | 2.816992 | down | 3.612477 | 4.232626 | 4.556055 | 3.092962 | 1.96008 | 2.865648 | chr1 | 1.23E+08 | 1.23E+08 | - |
| NONMMUT132449.1 |  | 0.013056 | 2.826434 | down | 5.161189 | 6.149645 | 5.946834 | 4.269105 | 3.931596 | 4.560019 | chr9 | 43036099 | 43036158 | + |
| NONMMUT069685.2 |  | 0.028908 | 2.436697 | up | 2.688005 | 1.69835 | 2.9597 | 3.710312 | 3.805323 | 3.685201 | chr9 | 66731605 | 66731664 | + |
| AK080003 | Cd274 | 0.0254 | 3.066021 | up | 4.056457 | 3.023039 | 2.637309 | 4.68179 | 5.236935 | 4.647182 | chr19 | 29444228 | 29444287 | + |
| NONMMUT101293.1 |  | 0.031542 | 2.45814 | up | 2.890312 | 1.815306 | 2.942956 | 4.040125 | 3.967157 | 3.533992 | chr17 | 63876198 | 63876257 | + |
| AK132561 |  | 0.030431 | 2.185994 | up | 2.577198 | 3.033704 | 3.619232 | 4.062359 | 4.533916 | 4.018727 | chr7 | 81551165 | 81551106 | - |
| NONMMUT085519.1 |  | 0.037551 | 2.065797 | down | 5.776574 | 6.014883 | 6.212032 | 5.042841 | 4.365356 | 5.455195 | chr11 | 78680569 | 78682607 | - |
| AK013093 | 2810414N06Rik | 0.041522 | 2.396439 | down | 8.67054 | 9.959509 | 9.911859 | 8.152028 | 8.351787 | 8.255418 | chr1 | 1.54E+08 | 1.54E+08 | - |
| NONMMUT086230.1 |  | 0.049296 | 2.114164 | up | 4.067589 | 2.990589 | 3.164544 | 4.662692 | 4.703864 | 4.096429 | chr12 | 8528476 | 8528535 | + |
| NONMMUT082660.1 |  | 0.037179 | 2.14507 | up | 5.173834 | 4.840571 | 4.86927 | 6.301349 | 6.497762 | 5.387638 | chr10 | 1.1E+08 | 1.1E+08 | - |
| NONMMUT083658.1 |  | 0.001114 | 2.20159 | up | 3.134746 | 2.776569 | 3.147396 | 4.14699 | 4.058431 | 4.268928 | chr11 | 53007566 | 53007625 | + |
| NONMMUT122690.1 |  | 0.006919 | 2.014162 | up | 3.1907 | 3.373186 | 3.80989 | 4.612384 | 4.411701 | 4.38023 | chr6 | 92736366 | 92736425 | + |
| NovelTID_00001515 |  | 0.042312 | 2.294287 | down | 3.913538 | 5.111589 | 4.987203 | 3.373064 | 3.285566 | 3.759562 | chr12 | 92791670 | 92791729 | + |
| AK077539 |  | 0.004231 | 2.013229 | up | 3.309097 | 3.576644 | 3.751511 | 4.670868 | 4.668748 | 4.32617 | chr6 | 50194957 | 50194898 | - |
| NONMMUT080006.1 |  | 0.041456 | 2.412216 | down | 7.291335 | 8.595987 | 8.556177 | 6.888205 | 6.903807 | 6.84041 | chr10 | 36869493 | 36869552 | + |
| NONMMUT093797.1 |  | 0.021324 | 2.009141 | down | 5.152535 | 5.139446 | 5.117107 | 4.350505 | 3.585401 | 4.453446 | chr14 | 1.24E+08 | 1.24E+08 | + |
| AK041536 |  | 0.032643 | 2.693694 | down | 7.501023 | 8.461227 | 8.846236 | 6.592065 | 6.69996 | 7.227704 | chr8 | 36442303 | 36442362 | + |
| TC1640282 |  | 8.26E-04 | 2.889462 | up | 2.839982 | 2.44172 | 2.539472 | 4.186717 | 3.911057 | 4.315802 | chr13 | 55604106 | 55604047 | - |
| NONMMUT060812.2 |  | 0.017539 | 3.280786 | up | 3.825764 | 2.992686 | 3.388562 | 4.401732 | 5.322937 | 5.624468 | chr7 | 37987656 | 38000711 | - |
| NovelTID_00002453 |  | 0.030187 | 2.924208 | down | 3.89728 | 5.334109 | 5.23167 | 3.12724 | 3.273285 | 3.418398 | chr15 | 93600413 | 93600472 | + |
| AK037738 | A130009I22Rik | 0.037456 | 2.321089 | up | 3.416885 | 2.584918 | 3.703453 | 4.66575 | 4.655951 | 4.02796 | chr8 | 71151458 | 71151399 | - |
| AK033243 | 8030474H12Rik | 0.006027 | 2.123433 | up | 3.005919 | 2.450244 | 2.408833 | 3.601865 | 3.836838 | 3.685489 | chr9 | 1.09E+08 | 1.09E+08 | - |
| AK020434 | 9430024C03Rik | 0.046333 | 2.810863 | down | 3.674282 | 5.174578 | 4.938379 | 2.637846 | 3.416096 | 3.260258 | chr10 | 88337749 | 88337690 | - |
| XR_393617 | LOC102631682 | 0.031692 | 3.130748 | down | 3.770017 | 4.516175 | 4.320064 | 2.221911 | 1.98627 | 3.458551 | chr11 | 1.1E+08 | 1.1E+08 | + |
| NONMMUT080699.1 |  | 0.041575 | 2.901924 | up | 3.94349 | 2.539969 | 2.546251 | 4.384793 | 4.259528 | 4.99642 | chr10 | 94416562 | 94416621 | + |
| NONMMUT108199.1 |  | 0.013897 | 2.161428 | down | 8.176912 | 8.902875 | 8.919426 | 7.684919 | 7.629086 | 7.349255 | chr2 | 1.57E+08 | 1.57E+08 | + |
| XR_883967 | BC051408 | 0.03697 | 2.497806 | up | 4.075881 | 2.617448 | 3.448539 | 4.829742 | 4.571001 | 4.703108 | chr18 | 28885285 | 28885344 | + |
| AK037309 |  | 0.027891 | 3.369819 | down | 6.043466 | 7.082311 | 6.957766 | 5.63992 | 4.941171 | 4.244436 | chr5 | 44234962 | 44235021 | + |
| XR_386591 | 4930554I06Rik | 4.21E-04 | 9.944571 | up | 1.098337 | 0.950846 | 1.177941 | 3.865031 | 4.90521 | 4.39861 | chr19 | 21179960 | 21180019 | + |
| NONMMUT126890.1 |  | 0.021561 | 3.427609 | down | 3.559883 | 4.1676 | 3.234457 | 2.645794 | 1.294593 | 1.689946 | chr7 | 66431525 | 66431466 | - |
| NONMMUT136951.1 |  | 0.030908 | 2.066465 | down | 8.378244 | 9.334252 | 9.208402 | 8.068809 | 8.008262 | 7.702333 | chrX | 55208067 | 55208008 | - |
| XR_387381 | LOC102632072 | 0.032742 | 2.118551 | up | 3.241617 | 2.948555 | 2.245246 | 3.977341 | 3.57931 | 4.128 | chr1 | 1.34E+08 | 1.34E+08 | + |
| AK135646 |  | 0.016119 | 2.173256 | up | 2.795942 | 3.147791 | 3.606417 | 4.510667 | 4.005608 | 4.393447 | chr15 | 51802508 | 51802449 | - |
| AK039568 |  | 0.041305 | 2.022006 | up | 7.455828 | 7.711626 | 7.802698 | 9.030296 | 8.966371 | 8.020846 | chr18 | 40529120 | 40529179 | + |
| AK041433 |  | 0.034092 | 2.098005 | down | 6.951615 | 7.976046 | 7.954396 | 6.54629 | 6.570155 | 6.558558 | chr5 | 1.23E+08 | 1.23E+08 | - |
| NONMMUT081769.1 |  | 0.015654 | 3.997387 | up | 2.099078 | 2.572299 | 1.029849 | 4.106333 | 3.514134 | 4.077931 | chr10 | 27827647 | 27855387 | - |
| NONMMUT115705.1 |  | 0.039847 | 2.605407 | down | 5.738884 | 7.116125 | 7.006372 | 4.982651 | 5.366778 | 5.367426 | chr4 | 1.31E+08 | 1.31E+08 | + |
| NONMMUT094861.1 |  | 0.036291 | 2.295568 | down | 8.285354 | 9.369751 | 9.446451 | 8.022768 | 7.707287 | 7.77495 | chr14 | 1.01E+08 | 1.01E+08 | - |
| AK041916 | B930012P20Rik | 0.036263 | 2.099895 | up | 5.037175 | 4.693779 | 4.861139 | 6.468122 | 6.006167 | 5.328756 | chr3 | 79036882 | 79036823 | - |
| NONMMUT095457.1 |  | 0.0109 | 5.097582 | down | 6.741134 | 6.451424 | 5.581161 | 4.575075 | 3.925748 | 3.223455 | chr15 | 25262196 | 25262255 | + |
| NONMMUT111826.1 |  | 0.021805 | 5.207823 | up | 4.984958 | 3.083964 | 3.819715 | 5.685686 | 6.487976 | 6.857015 | chr3 | 1.02E+08 | 1.02E+08 | + |
| NONMMUT067701.2 |  | 0.020231 | 2.206368 | down | 4.484376 | 4.886022 | 5.267437 | 4.14869 | 3.511699 | 3.552425 | chr8 | 1.27E+08 | 1.27E+08 | + |
| NONMMUT122911.1 |  | 0.040804 | 3.730467 | up | 3.381663 | 2.754222 | 2.884994 | 5.850262 | 5.099303 | 3.769382 | chr6 | 1.18E+08 | 1.18E+08 | + |
| NONMMUT112097.1 |  | 9.76E-04 | 6.914332 | down | 6.700781 | 7.289821 | 7.539245 | 4.79076 | 4.127626 | 4.24269 | chr3 | 1.24E+08 | 1.24E+08 | + |
| NONMMUT123605.1 |  | 0.001525 | 2.339393 | up | 2.315599 | 2.610827 | 2.767826 | 3.792641 | 3.942367 | 3.637647 | chr6 | 34962829 | 34962770 | - |
| AK043355 |  | 0.024467 | 2.273635 | up | 3.313304 | 2.829433 | 2.325221 | 4.333744 | 3.9722 | 3.717017 | chr2 | 58907350 | 58907291 | - |
| NONMMUT079149.1 |  | 0.018699 | 2.287667 | down | 3.95931 | 4.775327 | 4.584976 | 3.319256 | 3.534925 | 2.8838 | chr1 | 1.63E+08 | 1.63E+08 | - |
| NONMMUT102257.1 |  | 0.012844 | 2.638937 | up | 4.037085 | 3.304754 | 3.458451 | 4.553855 | 5.373032 | 5.073273 | chr17 | 56050278 | 56050087 | - |
| NONMMUT080044.1 |  | 0.00894 | 2.579011 | up | 2.779223 | 3.355759 | 3.345045 | 4.592604 | 4.125299 | 4.86258 | chr10 | 40543898 | 40543957 | + |
| NONMMUT124251.1 |  | 0.025055 | 2.218286 | up | 2.367015 | 2.861819 | 3.043578 | 4.416078 | 3.740827 | 3.563843 | chr6 | 1.08E+08 | 1.08E+08 | - |
| NONMMUT089027.1 |  | 0.025515 | 3.537848 | down | 4.305512 | 3.292226 | 3.146968 | 2.427118 | 1.729525 | 1.119445 | chr12 | 1.09E+08 | 1.09E+08 | - |
| NONMMUT094154.1 |  | 0.014846 | 2.315821 | up | 3.817297 | 3.291256 | 2.889496 | 4.406733 | 4.789615 | 4.436271 | chr14 | 26294956 | 26294897 | - |
| NONMMUT028028.2 |  | 0.016908 | 2.29156 | up | 2.630424 | 3.346268 | 2.77737 | 4.53566 | 3.904043 | 3.903348 | chr17 | 5720887 | 5720828 | - |
| NONMMUT101991.1 |  | 0.035117 | 2.166465 | down | 5.34353 | 5.826452 | 6.005677 | 5.19428 | 4.233971 | 4.401382 | chr17 | 31361882 | 31361823 | - |
| NONMMUT107764.1 |  | 0.040843 | 2.027491 | down | 4.189838 | 5.052992 | 4.899618 | 3.819499 | 3.274041 | 3.989822 | chr2 | 1.17E+08 | 1.17E+08 | + |
| NONMMUT079197.1 |  | 0.022981 | 4.110638 | up | 4.801409 | 3.190445 | 3.270715 | 5.432187 | 6.189998 | 5.758471 | chr1 | 1.67E+08 | 1.67E+08 | - |
| NONMMUT135177.1 |  | 0.001879 | 2.917862 | up | 2.462443 | 2.593224 | 2.065395 | 3.702687 | 3.871344 | 4.181768 | chr9 | 95428185 | 95428126 | - |
| AK167871 |  | 0.012934 | 2.042718 | up | 6.222409 | 5.499367 | 5.530896 | 6.818726 | 6.843658 | 6.68176 | chr12 | 1.07E+08 | 1.07E+08 | + |
| NONMMUT136499.1 |  | 0.038272 | 2.142726 | up | 2.299854 | 3.16624 | 3.328182 | 4.247083 | 3.697553 | 4.147981 | chrX | 1.48E+08 | 1.48E+08 | + |
| AK033130 |  | 0.016453 | 2.149943 | up | 3.65079 | 3.086016 | 2.734591 | 4.159792 | 4.412252 | 4.212248 | chr11 | 59507104 | 59507163 | + |
| AK143372 |  | 0.013983 | 2.103643 | up | 3.508827 | 2.99726 | 3.30517 | 4.491056 | 4.609261 | 3.929611 | chr10 | 8180191 | 8180132 | - |
| NONMMUT106987.1 |  | 0.022865 | 2.122155 | up | 2.912669 | 2.975192 | 3.707645 | 4.473156 | 3.962863 | 4.416078 | chr2 | 50307679 | 50307738 | + |
| NONMMUT131563.1 |  | 0.012541 | 2.35917 | up | 4.079252 | 3.362678 | 3.244834 | 4.577406 | 4.832756 | 4.99144 | chr8 | 1.1E+08 | 1.1E+08 | - |
| NONMMUT125608.1 |  | 0.018286 | 2.200277 | up | 3.760402 | 3.449487 | 3.424955 | 5.217509 | 4.526032 | 4.304359 | chr7 | 1.01E+08 | 1.01E+08 | + |
| NONMMUT130805.1 |  | 0.045473 | 3.374105 | down | 4.440711 | 3.947368 | 3.283424 | 2.87621 | 2.376665 | 1.155114 | chr8 | 50770636 | 50770577 | - |
| NONMMUT098003.1 |  | 0.017401 | 2.539901 | down | 5.873326 | 6.740775 | 6.555588 | 5.003004 | 4.685506 | 5.446862 | chr16 | 13470444 | 13470503 | + |
| NONMMUT101632.1 |  | 0.044594 | 2.106261 | down | 8.357698 | 9.422906 | 9.501909 | 8.029763 | 7.933034 | 8.095664 | chr17 | 93626616 | 93626675 | + |
| NONMMUT110768.1 |  | 0.003228 | 2.016179 | up | 2.927129 | 2.963703 | 3.356201 | 3.928495 | 4.172074 | 4.181335 | chr3 | 27788924 | 27788983 | + |
| NONMMUT120391.1 |  | 0.0036 | 2.297242 | up | 4.660946 | 4.166452 | 4.348872 | 5.335677 | 5.662436 | 5.777867 | chr5 | 51992044 | 51991985 | - |
| AK016022 | 4930542M03Rik | 0.002297 | 4.80779 | down | 4.459566 | 4.28339 | 4.498231 | 2.605482 | 2.310077 | 1.529508 | chr17 | 46016865 | 46016806 | - |
| AK037371 |  | 0.022183 | 2.265621 | up | 3.561068 | 3.473816 | 4.462182 | 5.053692 | 4.863255 | 5.11984 | chr9 | 24268383 | 24268324 | - |
| NONMMUT063242.2 |  | 0.017848 | 2.145315 | up | 3.914858 | 4.105433 | 4.286099 | 5.719504 | 5.031251 | 4.859202 | chr7 | 1.19E+08 | 1.19E+08 | + |
| AK078214 |  | 0.011506 | 2.157649 | up | 3.295377 | 2.773322 | 2.44117 | 3.980473 | 3.877303 | 3.980473 | chrX | 20377653 | 20377712 | + |
| NONMMUT025624.2 |  | 0.04172 | 2.005727 | down | 6.340897 | 7.261959 | 7.42221 | 6.055806 | 6.038699 | 5.918188 | chr16 | 15217310 | 15217251 | - |
| AK148710 |  | 0.00699 | 2.07282 | up | 3.848599 | 4.326709 | 3.64405 | 4.981171 | 5.065771 | 4.927201 | chr2 | 1.24E+08 | 1.24E+08 | + |
| NONMMUT105185.1 |  | 0.025508 | 3.038576 | up | 3.860363 | 2.678227 | 2.700128 | 4.296906 | 5.140974 | 4.611023 | chr19 | 15864051 | 15864110 | + |
| NONMMUT008412.2 |  | 0.036631 | 2.316635 | down | 5.510229 | 6.345446 | 6.272855 | 4.441961 | 5.392059 | 4.658415 | chr11 | 5345827 | 5345886 | + |
| AK089564 |  | 0.005328 | 2.2792 | up | 4.085059 | 3.723765 | 4.38135 | 5.403963 | 5.29546 | 5.056333 | chr1 | 36979706 | 36979647 | - |
| NONMMUT083571.1 |  | 0.012853 | 2.65637 | down | 5.029809 | 5.633308 | 5.326215 | 4.477363 | 3.601426 | 3.682174 | chr11 | 44825729 | 44825788 | + |
| ENSMUST00000127604.1 |  | 0.03389 | 2.009927 | down | 4.936774 | 4.66347 | 4.879734 | 3.665973 | 3.381953 | 4.410624 | chr11 | 43011083 | 43011039 | - |
| NONMMUT134941.1 |  | 0.02129 | 2.04429 | down | 4.587911 | 5.124417 | 5.533462 | 3.927089 | 4.120622 | 4.103282 | chr9 | 83574082 | 83574023 | - |
| AK049677 | 9930031P18Rik | 0.003115 | 2.005613 | up | 5.207267 | 5.03764 | 4.683981 | 5.917484 | 5.994625 | 6.028909 | chr13 | 1.09E+08 | 1.09E+08 | - |
| AK158273 |  | 3.32E-04 | 4.899092 | up | 1.559261 | 1.274278 | 1.85266 | 3.834223 | 3.67439 | 4.055128 | chr3 | 55696003 | 55696062 | + |
| NONMMUT130301.1 |  | 0.026098 | 2.44028 | up | 5.53179 | 4.657343 | 5.206207 | 5.877559 | 6.743403 | 6.635519 | chr8 | 33471687 | 33471628 | - |
| NONMMUT077743.1 |  | 0.023347 | 2.105035 | down | 7.512575 | 7.859254 | 8.042332 | 7.244876 | 6.500097 | 6.447656 | chr1 | 45953756 | 45953697 | - |
| CJ204422 |  | 0.041964 | 3.842356 | up | 3.147523 | 1.710888 | 0.961492 | 3.643801 | 4.153959 | 3.848117 | chr11 | 1.02E+08 | 1.02E+08 | + |
| NONMMUT075094.1 |  | 0.032909 | 2.559532 | down | 3.887694 | 4.337027 | 4.363601 | 2.593607 | 3.612807 | 2.314266 | chr1 | 19249697 | 19249756 | + |
| NONMMUT037057.2 |  | 0.042395 | 2.229658 | down | 7.876291 | 9.021353 | 9.029972 | 7.629582 | 7.506626 | 7.320943 | chr2 | 40854639 | 40854698 | + |
| NONMMUT082935.1 |  | 0.002322 | 2.139998 | up | 2.842423 | 3.099447 | 2.642237 | 4.101298 | 3.979137 | 3.796501 | chr10 | 1.25E+08 | 1.25E+08 | - |
| AK045227 |  | 0.015484 | 2.102086 | up | 3.520789 | 4.127024 | 3.997337 | 5.2919 | 4.934067 | 4.634649 | chr1 | 1.89E+08 | 1.89E+08 | - |
| NONMMUT137136.1 |  | 0.049905 | 2.029641 | down | 4.39803 | 4.998066 | 5.00896 | 3.467697 | 4.394804 | 3.478881 | chrX | 83042894 | 83042835 | - |
| NONMMUT106474.1 |  | 0.043841 | 2.226307 | up | 2.531237 | 3.65495 | 2.574189 | 4.34877 | 3.829189 | 4.046374 | chr19 | 59928052 | 59927993 | - |
| XR_384838 | LOC102639542 | 0.005506 | 2.187333 | down | 4.021228 | 4.257547 | 3.880345 | 2.698598 | 3.269707 | 2.803299 | chr16 | 37685002 | 37685061 | + |
| NONMMUT117752.1 |  | 0.02294 | 2.261302 | down | 5.887718 | 6.709005 | 6.466404 | 4.758834 | 5.272262 | 5.500571 | chr4 | 1.48E+08 | 1.48E+08 | - |
| NONMMUT079254.1 |  | 0.014465 | 7.512872 | down | 5.933249 | 5.312219 | 4.477018 | 1.77628 | 3.45867 | 1.759442 | chr1 | 1.7E+08 | 1.7E+08 | - |
| NONMMUT134122.1 |  | 0.011138 | 2.11156 | down | 4.899554 | 5.364338 | 5.406169 | 4.196744 | 3.812463 | 4.425926 | chr9 | 40724502 | 40724443 | - |
| AK019603 | 4930440F04Rik | 0.016505 | 2.467985 | down | 4.381546 | 5.254086 | 4.792215 | 3.862836 | 3.51949 | 3.13552 | chr12 | 1.09E+08 | 1.09E+08 | + |
| NONMMUT038394.2 |  | 0.008717 | 2.110472 | down | 4.729816 | 5.34614 | 5.119946 | 3.738864 | 4.021508 | 4.202834 | chr2 | 90510472 | 90510413 | - |
| NONMMUT020270.2 |  | 0.031535 | 2.318122 | down | 5.509211 | 6.507332 | 6.632468 | 5.046649 | 5.179014 | 4.784478 | chr14 | 32758864 | 32758923 | + |
| AK136376 |  | 0.042565 | 2.50562 | down | 4.781292 | 3.65578 | 3.556125 | 2.740366 | 2.258117 | 3.019212 | chr5 | 1.01E+08 | 1.01E+08 | - |
| NONMMUT119214.1 |  | 0.032691 | 3.285586 | up | 4.651741 | 3.168518 | 3.193637 | 4.981653 | 5.711309 | 5.469385 | chr5 | 1.14E+08 | 1.14E+08 | + |
| NONMMUT107547.1 |  | 0.008435 | 2.251784 | down | 5.559826 | 5.802647 | 5.925277 | 4.446755 | 5.018484 | 4.309306 | chr2 | 97876566 | 97935858 | + |
| NONMMUT082476.1 |  | 0.030654 | 2.174568 | down | 4.363741 | 4.342323 | 4.535412 | 2.879216 | 3.960161 | 3.039911 | chr10 | 95721356 | 95721297 | - |
| AK039391 |  | 0.031497 | 2.064736 | up | 3.977341 | 4.544232 | 3.971708 | 5.514373 | 5.424457 | 4.692324 | chr4 | 68575492 | 68575433 | - |
| ENSMUST00000200625.1 |  | 0.005446 | 2.166966 | up | 2.691109 | 3.046406 | 3.222726 | 4.286521 | 4.172274 | 3.848476 | chr3 | 34407714 | 34407773 | + |
| NR_040748 | Gm6639 | 0.002414 | 2.499016 | down | 4.138608 | 4.638356 | 4.459069 | 2.841299 | 3.172699 | 3.257955 | chr3 | 35536594 | 35536535 | - |
| NONMMUT128608.1 |  | 0.029124 | 2.430728 | down | 3.999015 | 3.541536 | 3.628977 | 2.899716 | 1.73518 | 2.690466 | chr8 | 48699555 | 48699614 | + |
| NONMMUT079416.1 |  | 0.01494 | 2.046898 | down | 3.994066 | 3.409257 | 3.398138 | 2.30416 | 2.546097 | 2.850886 | chr1 | 1.83E+08 | 1.83E+08 | - |
| AK020499 | 9430081G06Rik | 0.042257 | 2.404594 | down | 3.594252 | 4.092183 | 3.645051 | 2.591082 | 3.160772 | 1.782252 | chr13 | 35060378 | 35060437 | + |
| NONMMUT113638.1 |  | 0.003763 | 4.47329 | down | 5.937108 | 6.352273 | 5.837871 | 3.296721 | 4.401582 | 3.94494 | chr3 | 94608227 | 94608168 | - |
| AK136421 |  | 0.036797 | 2.292853 | up | 4.288333 | 3.269707 | 3.582102 | 4.567379 | 4.779639 | 5.384553 | chr1 | 78319542 | 78319601 | + |
| NONMMUT132788.1 |  | 0.031262 | 2.537685 | down | 4.005557 | 4.782025 | 5.218501 | 3.553366 | 2.902917 | 3.519262 | chr9 | 69928070 | 69928129 | + |
| NR_029384 | Otx2os1 | 0.042953 | 2.453826 | down | 4.458055 | 4.715229 | 3.335279 | 2.950086 | 2.625556 | 3.047822 | chr14 | 49412948 | 49413007 | + |
| NONMMUT117663.1 |  | 0.028875 | 2.14481 | down | 7.451863 | 8.326391 | 8.474994 | 7.021446 | 6.825001 | 7.10425 | chr4 | 1.42E+08 | 1.42E+08 | - |
| NONMMUT122003.1 |  | 0.032025 | 2.440443 | up | 4.067119 | 4.309749 | 3.878223 | 6.044757 | 5.337227 | 4.734535 | chr6 | 32372507 | 32372566 | + |
| NONMMUT078743.1 |  | 0.033349 | 2.026422 | down | 6.254064 | 6.607662 | 6.710092 | 4.930309 | 5.748449 | 5.836254 | chr1 | 1.27E+08 | 1.27E+08 | - |
| NONMMUT115842.1 |  | 0.041339 | 2.408909 | down | 4.622432 | 4.29701 | 4.386392 | 3.636478 | 3.528152 | 2.336066 | chr4 | 1.4E+08 | 1.4E+08 | + |
| NONMMUT103064.1 |  | 4.51E-04 | 2.993346 | up | 2.536694 | 2.325921 | 2.369291 | 4.248226 | 3.789942 | 3.939016 | chr18 | 25995666 | 25995725 | + |
| NONMMUT119537.1 |  | 0.006697 | 2.673244 | down | 4.668635 | 5.522375 | 5.37074 | 3.853735 | 3.608516 | 3.843725 | chr5 | 1.35E+08 | 1.35E+08 | + |
| NONMMUT111921.1 |  | 0.037301 | 2.020729 | down | 3.739692 | 4.23801 | 4.18531 | 2.665316 | 2.841882 | 3.611186 | chr3 | 1.08E+08 | 1.08E+08 | + |
| NONMMUT118845.1 |  | 0.027856 | 2.580449 | down | 4.612156 | 5.716989 | 5.758578 | 4.164644 | 4.12824 | 3.691971 | chr5 | 1.02E+08 | 1.02E+08 | + |
| NONMMUT008894.2 |  | 0.002285 | 3.772736 | down | 3.962618 | 3.722532 | 3.673816 | 2.155752 | 2.108825 | 1.347554 | chr11 | 23068663 | 23068722 | + |
| AK013746 | 2900064B16Rik | 0.038565 | 2.316952 | up | 2.801765 | 3.371916 | 2.962184 | 4.942033 | 4.116532 | 3.713985 | chr13 | 57745900 | 57745841 | - |
| NONMMUT126530.1 |  | 5.39E-05 | 2.088411 | down | 4.240999 | 4.442059 | 4.338827 | 3.291776 | 3.269106 | 3.273785 | chr7 | 25003798 | 25003739 | - |
| NONMMUT084129.1 |  | 0.049699 | 2.338905 | down | 3.815176 | 4.467448 | 5.007378 | 3.658911 | 3.242941 | 2.710649 | chr11 | 86823658 | 86823717 | + |
| AK086690 |  | 0.040583 | 2.001098 | down | 3.540185 | 3.856755 | 4.123074 | 3.278298 | 2.291449 | 2.947892 | chr5 | 21758946 | 21758887 | - |
| AK138315 |  | 0.037805 | 2.351178 | up | 2.863674 | 2.043379 | 3.410525 | 3.904231 | 3.970721 | 4.142777 | chr7 | 6846237 | 6846296 | + |
| NONMMUT123849.1 |  | 0.009417 | 2.216869 | down | 5.488648 | 5.66296 | 5.759009 | 4.238515 | 4.952011 | 4.274522 | chr6 | 64929543 | 64929484 | - |
| ENSMUST00000124085 | Zmynd8 | 0.005884 | 2.456483 | up | 3.433759 | 2.880219 | 3.467816 | 4.480041 | 4.84659 | 4.344946 | chr2 | 1.66E+08 | 1.66E+08 | - |
| NONMMUT129607.1 |  | 0.034336 | 2.091434 | down | 6.26965 | 7.362309 | 7.156038 | 5.879081 | 5.792463 | 5.922975 | chr8 | 1.16E+08 | 1.16E+08 | + |
| AK038790 |  | 0.006478 | 2.603109 | up | 2.197067 | 2.939965 | 2.86654 | 4.218754 | 4.108526 | 3.817 | chr11 | 21916135 | 21916076 | - |
| NONMMUT093479.1 |  | 0.009014 | 2.023969 | up | 4.737778 | 4.357547 | 4.055761 | 5.352446 | 5.564344 | 5.285857 | chr14 | 1.02E+08 | 1.02E+08 | + |
| NovelTID_00003273 |  | 0.006598 | 2.39712 | up | 2.046582 | 2.870456 | 2.36742 | 3.605822 | 3.7252 | 3.737342 | chr18 | 3000142 | 3000201 | + |

Abbreviations: AD: Alzheimer's disease; FC: fold change; C: control; E: experimental; ‘+’: sense strand of chromosome, ‘−’: antisense strand of chromosome.
