# Supplementary material for: Expression Profiles of Long Noncoding RNAs in Intranasal LPS-Mediated Alzheimer's Disease Model in Mice
Source: Biomed Res Int. 2019 Jan 21;2019:9642589. doi: 10.1155/2019/9642589 (PMC6369469; doi:10.1155/2019/9642589)
Supplement: Supplementary Materials — Supplementary Figure 1 (Figure s1). The hierarchical clustering of the 34 mRNAs related to neurons/nervous system diseases, inflammation, and olfactory pathway and the 24 lncRNAs coexpressed with the 34 mRNAs with the highest Pearson's correlation coefficients in AD and control hippocampal tissues. a. lncRNAs. b. mRNAs. Supplementary Figure 2 (Figure s2). lncRNA-mRNA-network analysis on 34 selected mRNAs and their coexpressed lncRNAs. Red arrow: lncRNAs; green round: mRNAs. The dotted lines between lncRNAs and mRNAs indicate a negative correlation, while the solid lines indicate a positive correlation. Supplementary Figure 3 (Figure s3) LncRNA-TFs Network of the selected 24 lncRNAs (the most 5 related lncRNA-TFs pairs according to the P value). Orange arrow: lncRNAs; blue diamonds: TFs. Supplementary Figure 4 (Figure s4) lncRNA-target-TFs network of the selected 24 lncRNAs (the most 5 related lncRNA-TFs pairs according to the P value). Orange arrow: lncRNAs; purple square: target mRNAs; Blue diamond: TFs. Supplementary File Table 1 Primers designed for qRT-PCR validation of candidate lncRNAs and mRNAs Supplementary File Table 2. The characters of differently expressed lncRNAs in AD and control. Supplementary File Table 3. The characters of differently expressed mRNAs in AD and control. Supplementary File Table 4. Characters of 34 mRNAs that related to neurons/nervous system diseases, inflammation, and olfactory pathway, and 24 lncRNAs that correlated with 34 mRNAs with the highest Pearson's correlation coefficients. Supplementary File Table 5. The KEGG pathways predicted for differently expressed mRNAs. [file 9642589.f1.zip › Table s3.doc]

Supplementary File Table 3 The characters of differently expressed mRNAs in AD and control.

| TargetID | GeneSymbol | p | FC (abs) | Regulation | C1.txt(normalized) | C2.txt(normalized) | C3.txt(normalized) | E2.txt(normalized) | E3.txt(normalized) | E4.txt(normalized) | chrom | start | end | strand |
| --- | --- | --- | --- | --- | --- | --- | --- | --- | --- | --- | --- | --- | --- | --- |
| NM_001033333 | Myrfl | 0.042279 | 2.352937 | down | 2.905605 | 4.144995 | 4.157411 | 2.590334 | 2.387509 | 2.52678 | chr10 | 1.16E+08 | 1.16E+08 | - |
| ENSMUST00000117520 | | 0.01264 | 2.212615 | down | 4.163802 | 4.092993 | 4.535743 | 3.482755 | 2.69789 | 3.174636 | chrX | 7527560 | 7527501 | - |
| ENSMUST00000117519 | | 0.011867 | 2.368384 | down | 2.248177 | 2.624563 | 3.066476 | 1.398985 | 1.67647 | 1.132052 | chr7 | 34566452 | 34566393 | - |
| NM_001113367 | Boll | 0.036446 | 2.675653 | down | 4.277601 | 4.130383 | 4.167458 | 1.864559 | 3.3185 | 3.132709 | chr1 | 55357012 | 55356953 | - |
| ENSMUST00000103336 | | 0.033391 | 2.10125 | down | 5.954882 | 6.178057 | 5.999852 | 4.550041 | 4.747047 | 5.621959 | chr6 | 68812318 | 68812259 | - |
| ENSMUST00000137938 | Itpr2 | 0.036616 | 2.968427 | down | 9.075708 | 10.41215 | 10.51566 | 8.646287 | 8.632458 | 8.015679 | chr6 | 1.46E+08 | 1.46E+08 | - |
| ENSMUST00000122109 | | 0.004744 | 2.003795 | down | 3.947516 | 3.543477 | 3.909125 | 2.557628 | 2.943521 | 2.890765 | chr11 | 40243282 | 40243341 | + |
| ENSMUST00000121515 | | 0.010625 | 2.95699 | down | 3.78293 | 4.385459 | 4.322039 | 2.647535 | 3.07248 | 2.078027 | chr2 | 45665249 | 45665308 | + |
| NAP113735-1 | | 0.008765 | 2.066005 | down | 4.411818 | 4.562617 | 4.664496 | 3.495408 | 3.858736 | 3.144255 | chr9 | 17537436 | 17537495 | + |
| ENSMUST00000120406 | | 0.0483 | 2.275585 | down | 4.691527 | 5.450468 | 5.545831 | 4.140054 | 4.549765 | 3.439293 | chr16 | 75772433 | 75772404 | - |
| NAP072303-1 | | 0.034179 | 2.872974 | down | 4.33919 | 5.721205 | 5.765317 | 3.847159 | 3.884518 | 3.526398 | chr1 | 1.5E+08 | 1.5E+08 | + |
| NM_010555 | Il1r2 | 0.005022 | 2.341135 | down | 4.251691 | 4.280327 | 4.762049 | 3.313115 | 3.3808 | 2.918529 | chr1 | 40182006 | 40182066 | + |
| NM_146310 | Olfr493 | 0.048387 | 2.183373 | down | 5.992756 | 6.408757 | 6.489833 | 5.643143 | 5.42828 | 4.440247 | chr7 | 1.15E+08 | 1.15E+08 | - |
| NM_001011864 | Olfr917 | 0.00868 | 2.095206 | down | 3.856444 | 4.423092 | 4.54825 | 3.320466 | 3.210846 | 3.095199 | chr9 | 38472872 | 38472813 | - |
| NM_011110 | Pla2g5 | 0.040511 | 2.097284 | down | 7.399104 | 7.761477 | 6.587297 | 6.140089 | 6.0545 | 6.347721 | chr4 | 1.38E+08 | 1.38E+08 | - |
| NM_053215 | Ugt2b37 | 0.007296 | 2.393793 | down | 6.933553 | 7.036206 | 7.35548 | 5.807119 | 6.241269 | 5.498957 | chr5 | 87669766 | 87669707 | - |
| NM_011474 | Sprr2h | 0.018708 | 3.121218 | down | 4.20804 | 4.323259 | 3.848235 | 1.787541 | 3.189889 | 2.475778 | chr3 | 92190899 | 92190958 | + |
| AK017561 | 5730414N17Rik | 0.01814 | 2.952239 | down | 9.55204 | 10.87212 | 10.40376 | 8.913403 | 8.499032 | 8.730059 | chr15 | 98704438 | 98704497 | + |
| NM_001205085 | Tbx20 | 0.004164 | 2.010418 | down | 3.861657 | 3.544146 | 3.933748 | 2.543932 | 2.962599 | 2.810535 | chr9 | 24563236 | 24563177 | - |
| NM_007739 | Col8a1 | 0.020354 | 2.21792 | down | 8.687661 | 8.936852 | 7.980413 | 7.224984 | 7.325808 | 7.60651 | chr16 | 57627092 | 57627033 | - |
| XM_011238190 | LOC102631585 | 0.014678 | 2.033978 | down | 4.144522 | 3.553173 | 3.322798 | 2.574868 | 2.640359 | 2.732353 |  |  |  |  |
| AK145733 | Arglu1 | 0.024624 | 2.189019 | down | 3.612807 | 4.060126 | 4.47048 | 3.172699 | 3.06896 | 2.510901 | chr8 | 8681913 | 8681854 | - |
| BG071781 | AU044856 | 0.040316 | 2.213825 | down | 3.409102 | 3.519143 | 3.481856 | 1.908334 | 1.975591 | 3.086552 | chr2 | 1.24E+08 | 1.24E+08 | + |
| ENSMUST00000173914 | Prc1 | 0.022809 | 2.13554 | down | 7.225196 | 7.873827 | 7.905883 | 6.302135 | 6.435786 | 6.983183 | chr7 | 87449197 | 87449256 | + |
| NM_173212 | Chrnb3 | 0.003638 | 2.200658 | down | 4.45109 | 4.706715 | 4.506991 | 3.166802 | 3.344247 | 3.739942 | chr8 | 28509397 | 28509456 | + |
| NM_011472 | Sprr2f | 0.03634 | 2.383154 | down | 4.378853 | 5.474484 | 5.579547 | 3.725281 | 4.142414 | 3.806574 | chr3 | 92170259 | 92170318 | + |
| NM_020564 | Sult5a1 | 0.036945 | 2.504483 | down | 8.112184 | 8.394837 | 8.4287 | 6.515967 | 6.624741 | 7.821471 | chr8 | 1.26E+08 | 1.26E+08 | - |
| DV653168 | C87487 | 0.022397 | 2.117301 | down | 3.83331 | 4.053853 | 4.151588 | 3.467362 | 2.501645 | 2.823064 | chr17 | 9416397 | 9416456 | + |
| NM_008205 | H2-M9 | 0.005729 | 2.337203 | down | 5.078754 | 5.651672 | 5.451611 | 3.940067 | 4.107871 | 4.459752 | chr17 | 36779522 | 36779232 | - |
| NM_025777 | Duoxa2 | 0.049245 | 2.490602 | down | 6.627897 | 6.809083 | 6.762821 | 4.713236 | 5.23259 | 6.304491 | chr2 | 1.22E+08 | 1.22E+08 | + |
| NM_146439 | Olfr937 | 0.038648 | 4.520608 | down | 3.721951 | 3.964179 | 4.07344 | 1.033598 | 1.033201 | 3.163219 | chr9 | 38868249 | 38868190 | - |
| NM_146918 | Olfr1180 | 0.046901 | 2.473512 | down | 4.948716 | 5.664109 | 5.606153 | 3.569846 | 3.848235 | 4.881215 | chr2 | 88252664 | 88252606 | - |
| NM_001195435 | 1190003K10Rik | 0.034878 | 2.669434 | down | 4.786937 | 5.670808 | 5.580638 | 3.22806 | 4.20804 | 4.352683 | chr13 | 64598922 | 64598863 | - |
| NM_001011837 | Olfr748 | 0.012369 | 2.122015 | down | 3.913873 | 4.152416 | 4.334219 | 3.122541 | 2.63654 | 3.385123 | chr14 | 51330634 | 51330693 | + |
| ENSMUST00000103667 | | 0.021059 | 2.456767 | down | 6.44417 | 6.733413 | 6.710173 | 4.881669 | 5.11895 | 5.996853 | chr14 | 54363320 | 54363379 | + |
| NM_011562 | Tdgf1 | 0.036368 | 2.268158 | down | 8.850992 | 8.337942 | 7.982327 | 6.753389 | 7.13558 | 7.737726 | chr9 | 1.11E+08 | 1.11E+08 | - |
| NM_001039095 | Glt6d1 | 0.019506 | 2.000237 | down | 4.903853 | 5.388912 | 5.740338 | 4.136038 | 4.408495 | 4.488056 | chr2 | 25649705 | 25649646 | - |
| BG066537 |  | 0.039107 | 2.059002 | down | 5.952904 | 6.853525 | 6.990459 | 5.785 | 5.450016 | 5.436036 | chr9 | 1.15E+08 | 1.15E+08 | + |
| NM_029974 | Dcst1 | 0.011625 | 2.045514 | down | 8.143517 | 8.431458 | 8.560554 | 7.299317 | 7.026747 | 7.712073 | chr3 | 89161823 | 89161764 | - |
| NM_013674 | Irf4 | 0.00491 | 2.035029 | down | 5.14852 | 5.697376 | 5.36942 | 4.351298 | 4.545148 | 4.243723 | chr13 | 30856460 | 30856519 | + |
| NM_001163730 | Gm9125 | 0.021696 | 4.256776 | down | 6.169517 | 7.217276 | 7.382996 | 4.215518 | 4.630751 | 5.654237 | chr3 | 93853003 | 93852944 | - |
| ENSMUST00000035501 | Loxhd1 | 0.041279 | 2.16031 | down | 6.961341 | 7.993044 | 8.024369 | 6.641257 | 6.722184 | 6.281601 | chr18 | 77620948 | 77621007 | + |
| NM_001105059 | Vmn2r62 | 0.037748 | 2.378556 | down | 7.833879 | 8.79406 | 9.022625 | 6.940057 | 7.400341 | 7.559907 | chr7 | 50019989 | 50019930 | - |
| NM_080852 | Slc7a12 | 0.011209 | 2.482019 | down | 6.127533 | 6.744471 | 6.475671 | 5.605545 | 4.888 | 4.91959 | chr3 | 14505556 | 14505615 | + |
| NM_182785 | Lypd4 | 0.014963 | 2.597331 | down | 3.554597 | 4.370606 | 3.939054 | 2.338617 | 3.058343 | 2.336207 | chr7 | 25649763 | 25649704 | - |
| BC008626 | Icam1 | 0.009406 | 2.113185 | down | 7.716296 | 8.343699 | 8.295526 | 6.870743 | 6.996696 | 7.249826 | chr9 | 20833186 | 20833245 | + |
| NM_177642 | Igfn1 | 0.010203 | 2.130995 | down | 4.714314 | 4.717207 | 4.072603 | 3.309097 | 3.301839 | 3.618606 | chr1 | 1.38E+08 | 1.38E+08 | - |
| NM_027157 | Krtap1-5 | 0.012641 | 2.521568 | down | 6.670304 | 7.624465 | 7.42354 | 5.686728 | 6.017679 | 6.01094 | chr11 | 99441448 | 99441389 | - |
| NM_001033177 | Krt76 | 0.021035 | 2.351411 | down | 4.0892 | 3.852928 | 3.802078 | 2.118457 | 3.235375 | 2.689794 | chr15 | 1.02E+08 | 1.02E+08 | - |
| NM_203492 | Mrgprg | 3.61E-04 | 4.18024 | down | 7.573424 | 7.538636 | 7.051352 | 5.411021 | 5.385064 | 5.176569 | chr7 | 1.51E+08 | 1.51E+08 | - |
| NM_001005485 | Olfr111 | 0.02071 | 2.42622 | down | 4.634961 | 5.163486 | 4.995305 | 3.789336 | 4.103788 | 3.064495 | chr17 | 37666993 | 37667052 | + |
| NM_145493 | Cplx4 | 0.044794 | 2.0393 | down | 7.625464 | 8.665537 | 8.700561 | 7.387369 | 7.210455 | 7.309516 | chr18 | 66116233 | 66116174 | - |
| NAP066544-1 | | 0.019656 | 2.018508 | down | 6.981952 | 7.58115 | 6.961429 | 6.497762 | 5.901924 | 6.084974 | chr3 | 26220966 | 26221025 | + |
| NM_134225 | Vmn1r193 | 0.042284 | 2.379163 | down | 4.684302 | 5.072371 | 5.122685 | 3.898087 | 4.291347 | 2.938562 | chr13 | 22311239 | 22311180 | - |
| NM_152944 | Mmp21 | 0.038616 | 2.380325 | down | 6.914256 | 8.024842 | 8.207559 | 6.426974 | 6.344897 | 6.621307 | chr7 | 1.41E+08 | 1.41E+08 | - |
| NAP072206-1 | | 0.007495 | 2.429918 | down | 9.548426 | 9.142878 | 8.994332 | 8.240778 | 8.025228 | 7.576906 | chr9 | 95434704 | 95434763 | + |
| NM_134131 | Tnfaip8 | 0.041535 | 2.51264 | down | 4.014356 | 4.300579 | 3.341743 | 1.918006 | 3.113976 | 2.637083 | chr18 | 50251297 | 50251356 | + |
| NM_001285788 | Ildr1 | 0.031042 | 2.22937 | down | 6.088433 | 7.006506 | 7.218567 | 5.466855 | 5.676522 | 5.700218 | chr16 | 36726213 | 36726272 | + |
| AK148871 | Ctnnd1 | 0.031392 | 2.18551 | down | 5.116544 | 6.204487 | 5.916805 | 4.859262 | 4.492367 | 4.502295 | chr2 | 84452052 | 84451993 | - |
| XM_006512713 | Utrn | 0.006089 | 2.117435 | down | 3.699136 | 3.410941 | 3.428314 | 2.593799 | 2.067746 | 2.629892 | chr10 | 12485097 | 12485038 | - |
| NM_146822 | Olfr640 | 0.006075 | 2.072088 | down | 4.20545 | 4.340774 | 3.953533 | 3.055717 | 2.868702 | 3.422083 | chr7 | 1.11E+08 | 1.11E+08 | - |
| NM_001079869 | Hoxb3 | 0.026099 | 2.162199 | down | 5.382208 | 5.80325 | 4.807015 | 4.436611 | 3.946544 | 4.271822 | chr11 | 96207114 | 96207173 | + |
| AK078619 | LOC382202 | 0.011517 | 2.192571 | down | 7.457811 | 8.005685 | 8.074062 | 6.810205 | 6.940232 | 6.389251 | chr15 | 74663606 | 74663547 | - |
| AK135377 |  | 0.02448 | 2.466004 | down | 3.633059 | 4.648556 | 3.91406 | 2.698305 | 3.159824 | 2.43102 | chr4 | 1.53E+08 | 1.53E+08 | - |
| AK036739 |  | 0.028586 | 2.062098 | down | 3.582102 | 3.954911 | 3.346829 | 2.081713 | 2.926702 | 2.743087 | chr1 | 59545386 | 59545327 | - |
| AK078387 | Gm15850 | 2.57E-04 | 2.20521 | down | 3.473593 | 3.470252 | 3.340642 | 2.230723 | 2.181398 | 2.449617 | chr1 | 1.38E+08 | 1.38E+08 | - |
| AK007900 | 1810058N15Rik | 0.016798 | 2.105348 | down | 4.11688 | 4.552007 | 4.516927 | 3.32799 | 2.913865 | 3.721782 | chr19 | 5561414 | 5561473 | + |
| XM_001481340 | Ssxa1 | 0.012129 | 4.180312 | down | 3.515703 | 3.360848 | 3.746745 | 1.023259 | 1.010492 | 2.398714 | chrX | 20699142 | 20699201 | + |
| AK033265 | Krt79 | 0.034756 | 2.097407 | down | 6.076806 | 5.530268 | 5.979421 | 4.206347 | 5.03018 | 5.144147 | chr15 | 1.02E+08 | 1.02E+08 | + |
| AW495160 |  | 0.03443 | 2.391564 | down | 3.635645 | 4.266829 | 3.694459 | 2.918386 | 2.984979 | 1.919706 | chr7 | 77492511 | 77492570 | + |
| XM_006532772 | Cdkl3 | 0.01583 | 2.318553 | down | 3.927529 | 3.887462 | 4.136701 | 2.719883 | 2.293033 | 3.299104 | chr11 | 51814512 | 51814571 | + |
| NM_145470 | Deptor | 0.029946 | 2.132896 | down | 9.584366 | 10.45188 | 10.43254 | 8.860026 | 8.935699 | 9.394618 | chr15 | 55090452 | 55090511 | + |
| AW487844 |  | 0.00286 | 2.157147 | down | 7.158094 | 7.430157 | 7.286986 | 5.976246 | 6.095044 | 6.476576 | chr17 | 34771089 | 34771030 | - |
| NM_026323 | Wfdc2 | 0.012134 | 2.598033 | down | 5.779483 | 6.037953 | 5.209326 | 4.201389 | 4.683738 | 4.009375 | chr2 | 1.64E+08 | 1.64E+08 | + |
| AK016243 | 4930458B22Rik | 0.035378 | 2.013382 | down | 8.59549 | 9.518547 | 9.520137 | 8.388795 | 8.057898 | 8.158619 | chr7 | 1.14E+08 | 1.14E+08 | - |
| NM_024290 | Tnfrsf23 | 0.024192 | 2.235594 | down | 3.938736 | 4.638882 | 4.431456 | 3.60778 | 2.725899 | 3.193419 | chr7 | 1.51E+08 | 1.51E+08 | - |
| XM_006540452 | LOC102640043 | 0.015998 | 2.070858 | down | 3.575184 | 3.908743 | 3.793377 | 3.167046 | 2.339617 | 2.619956 | chr7 | 4858116 | 4858057 | - |
| AK145165 | Faddos | 0.019439 | 2.179991 | down | 3.71464 | 3.411248 | 3.471508 | 2.428059 | 1.908959 | 2.887411 | chr7 | 1.52E+08 | 1.52E+08 | + |
| AK020087 | Gm41664 | 0.022517 | 2.716754 | down | 3.784009 | 4.42074 | 4.41203 | 2.156705 | 3.328754 | 2.805669 | chr18 | 6268327 | 6268386 | + |
| NM_001099318 | Rhox2c | 0.02745 | 2.011042 | up | 3.842003 | 3.490238 | 3.043126 | 4.208644 | 4.361646 | 4.828906 | chrX | 34868719 | 34868778 | + |
| XM_006519760 | Dnah12 | 0.047219 | 2.439374 | up | 4.060312 | 3.463481 | 2.504805 | 4.589091 | 4.691962 | 4.607079 | chr14 | 27537466 | 27537525 | + |
| XM_011251883 | LOC102641915 | 0.003928 | 2.096262 | up | 7.770742 | 7.701706 | 7.789213 | 8.972425 | 8.469823 | 9.022867 | chr3 | 1.46E+08 | 1.46E+08 | + |
| XM_011247688 | 4930519F16Rik | 0.023102 | 2.129319 | up | 2.337999 | 2.062396 | 1.445249 | 3.025775 | 3.307643 | 2.783403 | chrX | 1E+08 | 1E+08 | - |
| XM_011245236 | Dnah12 | 0.031269 | 2.159559 | up | 3.305472 | 3.621744 | 2.94043 | 4.623838 | 3.845524 | 4.730496 | chr14 | 27704799 | 27704858 | + |
| ENSMUST00000153974 | Edil3 | 0.01984 | 2.316515 | up | 3.336401 | 3.420221 | 3.661752 | 5.275357 | 4.240561 | 4.538324 | chr13 | 89272166 | 89272225 | + |
| AK030098 | Stk4 | 0.014892 | 2.111353 | up | 2.525627 | 3.187941 | 3.006003 | 4.280613 | 3.994895 | 3.678567 | chr2 | 1.64E+08 | 1.64E+08 | + |
| NM_177624 | Sntn | 0.03584 | 2.060802 | up | 3.259844 | 3.28647 | 2.591579 | 3.642324 | 4.493078 | 4.132108 | chr14 | 14514967 | 14515026 | + |
| AK045005 | Cblb | 0.011806 | 2.35068 | up | 4.086401 | 4.07788 | 4.008745 | 5.700516 | 5.416347 | 4.755396 | chr16 | 52050899 | 52050958 | + |
| NAP062643-1 | | 0.003085 | 2.481021 | up | 2.768667 | 2.233694 | 2.41559 | 4.047738 | 3.642146 | 3.660869 | chr7 | 25072552 | 25072493 | - |
| AK156706 | Gm11054 | 0.011922 | 2.239803 | up | 2.572096 | 2.139815 | 2.819973 | 3.546272 | 4.023199 | 3.45253 | chr14 | 76110920 | 76110979 | + |
| ENSMUST00000123019 | Tmem140 | 0.03948 | 2.663453 | up | 3.821348 | 3.637892 | 3.770734 | 5.633308 | 5.611961 | 4.2246 | chr6 | 34818899 | 34818958 | + |
| ENSMUST00000073309 | | 0.009128 | 2.003075 | up | 4.262028 | 3.79966 | 3.743514 | 5.204112 | 4.78099 | 4.826748 | chr14 | 27586844 | 27586903 | + |
| ENSMUST00000170237 | Ulk4 | 0.005661 | 2.716724 | up | 3.01787 | 2.404754 | 2.941501 | 3.909422 | 4.546895 | 4.233412 | chr9 | 1.21E+08 | 1.21E+08 | - |
| ENSMUST00000137124 | | 0.003438 | 3.822084 | up | 1.748402 | 2.442213 | 2.608983 | 4.481773 | 4.216548 | 3.904357 | chr11 | 57877442 | 57877383 | - |
| AK164100 | Senp7 | 0.017129 | 2.435905 | up | 3.781083 | 2.838722 | 3.03045 | 4.796985 | 4.269038 | 4.437607 | chr16 | 56099522 | 56099581 | + |
| AK169474 | Hectd1 | 3.70E-04 | 2.054047 | up | 3.576482 | 3.584037 | 3.844137 | 4.743321 | 4.64533 | 4.731413 | chr12 | 52859144 | 52859085 | - |
| ENSMUST00000185525 | Fam135a | 0.003522 | 2.104088 | up | 3.771844 | 3.450086 | 3.988958 | 4.91359 | 4.855847 | 4.661035 | chr1 | 24058953 | 24058894 | - |
| NM_010544 | Ihh | 0.014141 | 2.455252 | up | 3.825513 | 3.255953 | 2.988757 | 4.806343 | 4.274632 | 4.876861 | chr1 | 74991965 | 74991906 | - |
| NM_029305 | 1700003F12Rik | 0.011403 | 2.179192 | up | 2.710013 | 3.314917 | 2.598352 | 4.240428 | 3.861876 | 3.892359 | chr2 | 1.54E+08 | 1.54E+08 | + |
| XM_011244607 | Gm10760 | 0.025304 | 2.194011 | up | 7.276891 | 6.517456 | 6.521952 | 8.309148 | 7.772643 | 7.635222 | chr13 | 76521826 | 76521767 | - |
| NM_028798 | Crct1 | 0.047367 | 5.425421 | up | 3.810026 | 1.862859 | 0.960686 | 4.272837 | 4.889685 | 4.790255 | chr3 | 92818251 | 92818192 | - |
| ENSMUST00000118959 | Fanci | 0.034278 | 4.774995 | up | 4.99599 | 2.881154 | 3.28212 | 5.442463 | 6.479323 | 6.003974 | chr7 | 86554409 | 86554468 | + |
| NM_019941 | Zfp235 | 1.48E-04 | 2.125239 | up | 2.523011 | 2.696019 | 2.728806 | 3.659341 | 3.810479 | 3.740891 | chr7 | 24928153 | 24928212 | + |
| ENSMUST00000115299 | Kcnq5 | 0.015848 | 2.107933 | up | 4.278879 | 3.82362 | 4.566064 | 5.60902 | 5.095182 | 5.191847 | chr1 | 21456385 | 21456326 | - |
| ENSMUST00000140071 | Glt8d2 | 0.022507 | 2.217162 | up | 3.153423 | 3.819953 | 2.803299 | 4.235593 | 4.373569 | 4.613656 | chr10 | 82132083 | 82132024 | - |
| AK047982 | Rtn4 | 0.005346 | 2.161254 | up | 4.602896 | 4.431994 | 4.664656 | 6.01284 | 5.667381 | 5.35493 | chr11 | 29597386 | 29597445 | + |
| XM_006496600 | Mndal | 0.038736 | 2.149548 | up | 4.267289 | 4.495994 | 4.116347 | 6.089222 | 5.099439 | 5.003067 | chr1 | 1.76E+08 | 1.76E+08 | - |
| AK079715 |  | 0.036399 | 2.517263 | up | 3.857283 | 4.58317 | 3.499271 | 5.807364 | 5.321559 | 4.80637 | chr1 | 1.76E+08 | 1.76E+08 | - |
| AK082313 |  | 0.008204 | 2.169656 | up | 2.84765 | 3.450717 | 2.726061 | 4.028425 | 4.164309 | 4.184094 | chr19 | 24240988 | 24240929 | - |
| XM_006501463 | Ankub1 | 0.030101 | 2.091502 | up | 6.52569 | 6.294697 | 5.469768 | 7.210406 | 7.078633 | 7.194734 | chr3 | 57461262 | 57461203 | - |
| XM_006540228 | Sae1 | 0.011769 | 2.0142 | up | 4.030104 | 4.265405 | 4.118833 | 5.437135 | 5.290389 | 4.717439 | chr7 | 16932506 | 16932447 | - |
| AK085353 | D630014O11Rik | 0.024771 | 2.15415 | up | 2.748826 | 3.003053 | 3.754651 | 4.425246 | 4.296049 | 4.106591 | chr17 | 73987464 | 73987405 | - |
| AK033078 | Dleu2 | 0.002183 | 2.513863 | up | 3.328326 | 2.833032 | 2.74807 | 4.413803 | 4.251512 | 4.233832 | chr14 | 62237336 | 62237277 | - |
| AK040039 |  | 0.013679 | 2.155998 | up | 2.353324 | 2.625556 | 3.184291 | 4.01331 | 3.674744 | 3.800183 | chr16 | 24770289 | 24770348 | + |
| AK088368 | E430014B02Rik | 0.022015 | 2.163547 | up | 2.502808 | 1.998227 | 3.044586 | 3.555464 | 3.608259 | 3.722093 | chr17 | 51884923 | 51884864 | - |
| AK032194 |  | 0.047209 | 2.867978 | up | 5.202869 | 3.740193 | 3.651067 | 5.720211 | 6.040276 | 5.393745 | chr7 | 1.04E+08 | 1.04E+08 | - |
| ENSMUST00000188949 | | 0.001154 | 2.80254 | up | 4.703066 | 4.665091 | 4.799329 | 5.871367 | 6.300771 | 6.455551 | chr1 | 87827700 | 87827759 | + |
| NAP092468-001 | | 0.021661 | 2.135387 | up | 5.162616 | 4.732514 | 4.565928 | 5.458141 | 6.276118 | 6.010291 | chr12 | 3521887 | 3521828 | - |
| ENSMUST00000173598 | Chadl | 0.03754 | 2.80381 | up | 5.426766 | 4.611343 | 4.017044 | 5.742748 | 6.647915 | 6.126656 | chr15 | 81512634 | 81512575 | - |
| XM_006514426 | LOC102637507 | 0.007865 | 9.528819 | up | 2.995747 | 0.973698 | 2.010578 | 4.65939 | 5.381611 | 5.695915 | chr10 | 1.02E+08 | 1.02E+08 | + |
| AK002931 | 0710001A04Rik | 0.034208 | 2.719923 | up | 5.113728 | 3.993049 | 3.644373 | 5.554561 | 5.913213 | 5.614076 | chr18 | 25877572 | 25877631 | + |
| AK005987 | 1700015C17Rik | 0.015297 | 2.188358 | up | 3.671804 | 4.154001 | 4.166654 | 5.47706 | 5.198529 | 4.706414 | chr3 | 1.53E+08 | 1.53E+08 | - |

Abbreviations: AD: Alzheimer's disease; FC: fold change; C: control; E: experimental; ‘+’: sense strand of chromosome, ‘−’: antisense strand of chromosome.
