# Supplementary material for: Expression Profiles of Long Noncoding RNAs in Intranasal LPS-Mediated Alzheimer's Disease Model in Mice
Source: Biomed Res Int. 2019 Jan 21;2019:9642589. doi: 10.1155/2019/9642589 (PMC6369469; doi:10.1155/2019/9642589)
Supplement: Supplementary Materials — Supplementary Figure 1 (Figure s1). The hierarchical clustering of the 34 mRNAs related to neurons/nervous system diseases, inflammation, and olfactory pathway and the 24 lncRNAs coexpressed with the 34 mRNAs with the highest Pearson's correlation coefficients in AD and control hippocampal tissues. a. lncRNAs. b. mRNAs. Supplementary Figure 2 (Figure s2). lncRNA-mRNA-network analysis on 34 selected mRNAs and their coexpressed lncRNAs. Red arrow: lncRNAs; green round: mRNAs. The dotted lines between lncRNAs and mRNAs indicate a negative correlation, while the solid lines indicate a positive correlation. Supplementary Figure 3 (Figure s3) LncRNA-TFs Network of the selected 24 lncRNAs (the most 5 related lncRNA-TFs pairs according to the P value). Orange arrow: lncRNAs; blue diamonds: TFs. Supplementary Figure 4 (Figure s4) lncRNA-target-TFs network of the selected 24 lncRNAs (the most 5 related lncRNA-TFs pairs according to the P value). Orange arrow: lncRNAs; purple square: target mRNAs; Blue diamond: TFs. Supplementary File Table 1 Primers designed for qRT-PCR validation of candidate lncRNAs and mRNAs Supplementary File Table 2. The characters of differently expressed lncRNAs in AD and control. Supplementary File Table 3. The characters of differently expressed mRNAs in AD and control. Supplementary File Table 4. Characters of 34 mRNAs that related to neurons/nervous system diseases, inflammation, and olfactory pathway, and 24 lncRNAs that correlated with 34 mRNAs with the highest Pearson's correlation coefficients. Supplementary File Table 5. The KEGG pathways predicted for differently expressed mRNAs. [file 9642589.f1.zip › Table s5.doc]

Supplementary File Table 5 The KEGG pathways predicted for differently expressed mRNAs

| Term_ID | Term_description | GeneSymbols | P_value |
| --- | --- | --- | --- |
| path:mmu04514 | Cell adhesion molecules (CAMs) | Icam1;H2-M9;Cd274 | 0.013637 |
| path:mmu04918 | Thyroid hormone synthesis | Itpr2;Duoxa2 | 0.021484 |
| path:mmu05416 | Viral myocarditis | Icam1;H2-M9 | 0.026024 |
| path:mmu04972 | Pancreatic secretion | Itpr2;Pla2g5 | 0.040994 |
| path:mmu04725 | Cholinergic synapse | Itpr2;Kcnq5 | 0.049067 |
| path:mmu04670 | Leukocyte transendothelial migration | Icam1;Ctnnd1 | 0.054484 |
| path:mmu05166 | HTLV-I infection | Il1r2;Icam1;H2-M9 | 0.055675 |
| path:mmu04270 | Vascular smooth muscle contraction | Itpr2;Pla2g5 | 0.060924 |
| path:mmu04120 | Ubiquitin mediated proteolysis | Sae1;Cblb | 0.072791 |
| path:mmu00592 | alpha-Linolenic acid metabolism | Pla2g5 | 0.07724 |
| path:mmu00053 | Ascorbate and aldarate metabolism | Ugt2b37 | 0.082939 |
| path:mmu05143 | African trypanosomiasis | Icam1 | 0.105398 |
| path:mmu00040 | Pentose and glucuronate interconversions | Ugt2b37 | 0.108168 |
| path:mmu04740 | Olfactory transduction | Olfr640;Olfr748;Olfr111;  Olfr493;Olfr937;Olfr1180 | 0.111168 |
| path:mmu04975 | Fat digestion and absorption | Pla2g5 | 0.119166 |
| path:mmu00860 | Porphyrin and chlorophyll metabolism | Ugt2b37 | 0.121895 |
| path:mmu00565 | Ether lipid metabolism | Pla2g5 | 0.130034 |
| path:mmu05205 | Proteoglycans in cancer | Itpr2;Cblb | 0.133715 |
| path:mmu05144 | Malaria | Icam1 | 0.143439 |
| path:mmu04340 | Hedgehog signaling pathway | Ihh | 0.143439 |
| path:mmu05169 | Epstein-Barr virus infection | Icam1;H2-M9 | 0.14426 |
| path:mmu00591 | Linoleic acid metabolism | Pla2g5 | 0.146096 |
| path:mmu00983 | Drug metabolism - other enzymes | Ugt2b37 | 0.148745 |
| path:mmu05150 | Staphylococcus aureus infection | Icam1 | 0.148745 |
| path:mmu03460 | Fanconi anemia pathway | Fanci | 0.148745 |
| path:mmu00500 | Starch and sucrose metabolism | Ugt2b37 | 0.156645 |
| path:mmu04014 | Ras signaling pathway | Stk4;Pla2g5 | 0.159321 |
| path:mmu05223 | Non-small cell lung cancer | Stk4 | 0.161873 |
| path:mmu05330 | Allograft rejection | H2-M9 | 0.161873 |
| path:mmu05332 | Graft-versus-host disease | H2-M9 | 0.167069 |
| path:mmu04730 | Long-term depression | Itpr2 | 0.174807 |
| path:mmu04721 | Synaptic vesicle cycle | Cplx4 | 0.17737 |
| path:mmu04940 | Type I diabetes mellitus | H2-M9 | 0.179926 |
| path:mmu00980 | Metabolism of xenobiotics by cytochrome P450 | Ugt2b37 | 0.182475 |
| path:mmu04010 | MAPK signaling pathway | Il1r2;Stk4 | 0.185808 |
| path:mmu00982 | Drug metabolism - cytochrome P450 | Ugt2b37 | 0.187549 |
| path:mmu04720 | Long-term potentiation | Itpr2 | 0.190075 |
| path:mmu05320 | Autoimmune thyroid disease | H2-M9 | 0.200103 |
| path:mmu04924 | Renin secretion | Itpr2 | 0.202591 |
| path:mmu05220 | Chronic myeloid leukemia | Cblb | 0.205072 |
| path:mmu04971 | Gastric acid secretion | Itpr2 | 0.207546 |
| path:mmu04520 | Adherens junction | Ctnnd1 | 0.207546 |
| path:mmu04144 | Endocytosis | H2-M9;Cblb | 0.212934 |
| path:mmu04970 | Salivary secretion | Itpr2 | 0.217366 |
| path:mmu05100 | Bacterial invasion of epithelial cells | Cblb | 0.217366 |
| path:mmu05323 | Rheumatoid arthritis | Icam1 | 0.22707 |
| path:mmu04612 | Antigen processing and presentation | H2-M9 | 0.229478 |
| path:mmu04640 | Hematopoietic cell lineage | Il1r2 | 0.234272 |
| path:mmu04012 | ErbB signaling pathway | Cblb | 0.239038 |
| path:mmu04540 | Gap junction | Itpr2 | 0.239038 |
| path:mmu00140 | Steroid hormone biosynthesis | Ugt2b37 | 0.239038 |
| path:mmu00830 | Retinol metabolism | Ugt2b37 | 0.243775 |
| path:mmu00590 | Arachidonic acid metabolism | Pla2g5 | 0.243775 |
| path:mmu04912 | GnRH signaling pathway | Itpr2 | 0.243775 |
| path:mmu05204 | Chemical carcinogenesis | Ugt2b37 | 0.250828 |
| path:mmu00564 | Glycerophospholipid metabolism | Pla2g5 | 0.255494 |
| path:mmu04070 | Phosphatidylinositol signaling system | Itpr2 | 0.262443 |
| path:mmu04915 | Estrogen signaling pathway | Itpr2 | 0.26704 |
| path:mmu04064 | NF-kappa B signaling pathway | Icam1 | 0.27161 |
| path:mmu04922 | Glucagon signaling pathway | Itpr2 | 0.276153 |
| path:mmu04723 | Retrograde endocannabinoid signaling | Itpr2 | 0.278414 |
| path:mmu04660 | T cell receptor signaling pathway | Cblb | 0.280668 |
| path:mmu04668 | TNF signaling pathway | Icam1 | 0.291839 |
| path:mmu04114 | Oocyte meiosis | Itpr2 | 0.302844 |
| path:mmu04724 | Glutamatergic synapse | Itpr2 | 0.305025 |
| path:mmu05146 | Amoebiasis | Il1r2 | 0.311529 |
| path:mmu04650 | Natural killer cell mediated cytotoxicity | Icam1 | 0.311529 |
| path:mmu04750 | Inflammatory mediator regulation of TRP channels | Itpr2 | 0.32859 |
| path:mmu04611 | Platelet activation | Itpr2 | 0.339047 |
| path:mmu04726 | Serotonergic synapse | Itpr2 | 0.343185 |
| path:mmu04068 | FoxO signaling pathway | Stk4 | 0.343185 |
| path:mmu04728 | Dopaminergic synapse | Itpr2 | 0.347299 |
| path:mmu05162 | Measles | Cblb | 0.349347 |
| path:mmu05200 | Pathways in cancer | Stk4;Cblb | 0.351471 |
| path:mmu04910 | Insulin signaling pathway | Cblb | 0.359494 |
| path:mmu04630 | Jak-STAT signaling pathway | Cblb | 0.38517 |
| path:mmu04921 | Oxytocin signaling pathway | Itpr2 | 0.392869 |
| path:mmu05164 | Influenza A | Icam1 | 0.415418 |
| path:mmu04022 | cGMP-PKG signaling pathway | Itpr2 | 0.417261 |
| path:mmu04145 | Phagosome | H2-M9 | 0.420929 |
| path:mmu05010 | Alzheimer's disease | Itpr2 | 0.42639 |
| path:mmu05202 | Transcriptional misregulation in cancer | Il1r2 | 0.42639 |
| path:mmu04020 | Calcium signaling pathway | Itpr2 | 0.433594 |
| path:mmu05016 | Huntington's disease | Dnah12 | 0.464954 |
| path:mmu05168 | Herpes simplex infection | H2-M9 | 0.48165 |
| path:mmu04015 | Rap1 signaling pathway | Ctnnd1 | 0.489809 |
| path:mmu05203 | Viral carcinogenesis | H2-M9 | 0.518177 |
| path:mmu04060 | Cytokine-cytokine receptor interaction | Il1r2 | 0.564917 |
| path:mmu04080 | Neuroactive ligand-receptor interaction | Chrnb3 | 0.590578 |
| path:mmu01100 | Metabolic pathways | Pla2g5;Ugt2b37 | 0.924115 |

Abbreviations: KEGG : Kyoto Encyclopedia of Genes and Genomes.
